# Contribution of pectin-degrading bacteria to the quality of cigar fermentation: an analysis based on microbial communities and physicochemical components

**DOI:** 10.3389/fmicb.2024.1481158

**Published:** 2024-11-14

**Authors:** Youbo Su, Yonghe Cui, Kejian Fu, Lingduo Bu, Yucui Sun, Qi Zhou, Yuming Yin, Yulong Sun, Huating Yang, Lang Wu, Xueru Song

**Affiliations:** ^1^College of Resources and Environment, Yunnan Agricultural University, Kunming, China; ^2^Yunnan Tobacco Company, Yuxi City Corporation, Yuxi, China; ^3^Yunnan Yuntianhua Co., Ltd., Research and Development Center, Kunming, China

**Keywords:** cigar, fermentation, pectin-degrading bacteria, physicochemical properties, microbial community

## Abstract

**Objective:**

This study aims to investigate the effects of pectin-degrading bacteria on the microbial community and physicochemical properties during the fermentation process of cigar tobacco, evaluating its potential in reducing green bitterness and enhancing aroma.

**Methods:**

By isolating and screening pectin-degrading bacteria, high-throughput sequencing and physicochemical analysis were employed to compare the microbial flora and physicochemical component differences in different treatment groups of cigar tobacco. Furthermore, correlation analysis was performed to examine the relationships between these variables.

**Results:**

The results showed that the strains YX-2 and DM-3, isolated from the cigar tobacco variety “*Yunxue No. 1*,” exhibited strong pectin-degrading abilities. Phylogenetic analysis revealed that strain YX-2 is highly homologous to *Bacillus flexus*, while strain DM-3 is highly homologous to *Bacillus siamensis*. After fermentation, the addition of strains YX-2 and DM-3 significantly reduced the pectin content in the tobacco leaves, increased the total sugar and reducing sugar content, reduced green bitterness, and markedly enhanced the total aroma components. Notably, DM-3 exhibited outstanding performance in the production of Maillard reaction products. Microbial community analysis showed that the addition of pectin-degrading bacteria significantly increased the diversity of both bacteria and fungi, especially in the TDM3 group, where the relative abundance of *Pseudomonas* was notably elevated. Correlation analysis revealed that *Pseudomonas* had a significant positive correlation with both reducing sugar and total sugar, and a significant negative correlation with pectin, indicating its important role in sugar metabolism and pectin degradation. Additionally, fungal genera such as *Cercospora* were significantly negatively correlated with total sugar and total nitrogen, while *Eurotium* was closely associated with pectin degradation and reducing sugar accumulation.

**Conclusion:**

This study found that the addition of Pectin-degrading bacteria YX-2 and DM-3 significantly optimized the microbial community structure during the cigar tobacco fermentation process and improved the physicochemical properties of the tobacco leaves, with notable effects in reducing green bitterness and enhancing aroma.

## 1 Introduction

Cigars are a premium tobacco product, renowned for their unique flavor, rich aroma, and complex taste. Unlike regular cigarettes, the production process of cigars is more intricate and refined. Crafting a cigar involves several detailed steps, from cultivation to the final product, with each stage being crucial. The main processes include tobacco leaf cultivation and harvesting, drying, fermentation, rolling, and finished cigar storage, with fermentation being the key step in enhancing quality. Fermentation is not only a physical transformation but also a process of chemical and biological changes. During fermentation, complex organic substances in the tobacco leaves, such as carbohydrates, proteins, starches, cellulose, and fats, are decomposed and transformed by microorganisms and enzymes, producing a variety of aromatic substances and flavor compounds (Si et al., 2023; Li et al., 2020; Banožić et al., 2020). These aromatic substances, including carotenoid degradation products, chlorophyll degradation products, and Maillard reaction products, impart the cigar with its distinctive aroma and complex flavor, clearly distinguishing it from regular cigarettes (Fu et al., 2024; Rodriguez-Bustamante et al., 2005). Fresh tobacco leaves contain some undesirable odors and irritants, such as ammonia, nicotine, and various phenolic compounds. During fermentation, these substances are degraded or transformed into milder compounds by microbial activity, significantly reducing the harshness and unpleasant odors of the tobacco leaves (Zhang et al., 2024; Ning et al., 2023). This process not only enhances the smoking experience but also results in smoke that is smoother and sweeter during combustion. Furthermore, fermentation can alter the physical structure of the tobacco leaves, making them softer and more elastic, which is more suitable for cigar rolling. During fermentation, macromolecules such as pectin and cellulose in the tobacco leaves are partially degraded, leading to changes in the leaf tissue structure. These changes increase flexibility and rollability. This transformation not only facilitates the rolling of high-quality cigars but also improves the uniformity and durability of the cigar’s burn during smoking (Dai et al., 2020). Most importantly, fermentation can reduce the levels of certain harmful substances in the tobacco leaves. Through fermentation, the safety of cigars is improved, as harmful substances like nitrates, nitrites, and heavy metals are metabolized or transformed by microorganisms, thereby reducing their potential health risks (Li et al., 2023; Ning et al., 2023). In summary, the fermentation process is critical in enhancing aroma and flavor, eliminating undesirable odors and irritants, improving physical properties, reducing harmful substances, and stabilizing chemical composition. These improvements not only elevate the quality and smoking experience of cigars but also provide a strong competitive edge in the market.

Pectin is a complex polysaccharide, primarily composed of galacturonic acid units linked by *α*-1,4-glycosidic bonds, and is widely present in plant cell walls (Thakur et al., 1997; Mohnen, 2008). In tobacco leaves, pectin is a major component of the cell wall and intercellular matrix, with its content and degradation directly influencing the leaves’ physical and chemical properties. During fermentation, pectin is broken down by microorganisms and enzymes into various low-molecular-weight compounds, such as galacturonic acid, oligosaccharides, and other organic acids, which significantly impact the taste of tobacco leaves (Bedzo et al., 2023; Kratchanova et al., 1995). Organic acids, such as galacturonic acid, produced during pectin degradation, are the main sources of green bitterness. Their high acidity directly affects the tobacco leaves’ flavor, imparting a strong green bitterness (Weng et al., 2024). For example, galacturonic acid can metabolize into simple organic acids like formic acid and acetic acid, which, in high concentrations, impart a strong bitterness. Additionally, microbial metabolism during pectin degradation can produce aldehydes and ketones, compounds with pungent odors and bitter tastes, further negatively affecting the tobacco’s sensory quality (Yang et al., 2022). When fermentation is incomplete or poorly controlled, these bitter compounds can accumulate, intensifying the green bitterness of the tobacco leaves. Pectin degradation may also generate volatile organic compounds, such as methanol and ethanol, which, at high concentrations, can negatively impact the aroma and taste of tobacco leaves, increasing the perception of green bitterness (Zhu et al., 2014; Zhao et al., 2024). If pectin content is too high and not fully degraded during fermentation, the remaining pectin can continue to release bitter compounds, leading to strong green bitterness during smoking. Moreover, high pectin content can result in uneven microbial distribution and activity during fermentation, leading to incomplete pectin degradation in some areas, causing localized intense green bitterness (Zhang et al., 2024; Song et al., 2024). Therefore, effective fermentation management and techniques are required during cigar fermentation to significantly reduce pectin content and minimize green bitterness, thereby improving the overall quality of the tobacco leaves.

Pectin-degrading bacteria are microorganisms capable of efficiently degrading pectin, and they hold significant potential for application in cigar fermentation. By introducing pectin-degrading bacteria, the pectin content in tobacco leaves can be significantly reduced, thereby decreasing bitterness and astringency while enhancing the aroma and flavor of the tobacco. These strains rapidly secrete large amounts of pectinases, including pectin lyase and polygalacturonase, which quickly break down pectin into monosaccharides and oligosaccharides, reducing the formation of bitter compounds (Weng et al., 2024; Rehman et al., 2015). Furthermore, pectin-degrading bacteria exhibit strong adaptability under various fermentation conditions, maintaining activity in high-salt, low-pH, and fluctuating temperature environments, ensuring the stability and continuity of the fermentation process (Zhao et al., 2024; Nograšek et al., 2015). In addition to pectinases, some pectin-degrading bacteria also secrete other degradative enzymes, such as cellulases and xylanases, which synergistically act on the complex carbohydrates in tobacco leaves, further improving their physical and sensory qualities (Zheng et al., 2017; Bonnin et al., 2014). Moreover, pectin-degrading bacteria can inhibit the growth of harmful microorganisms through competitive inhibition, reducing the production of harmful metabolites and further enhancing the quality of fermented tobacco leaves (Li et al., 2024; Soriano et al., 2005). Therefore, effective fermentation management and the addition of pectin-degrading bacteria during cigar fermentation can significantly reduce pectin content, lessen green bitterness, and enhance the overall quality of tobacco leaves. This approach offers new strategies for optimizing fermentation and improving product quality.

In this study, high-quality healthy cigar tobacco leaves were used to screen for pectin-degrading bacteria, which were then applied in cigar fermentation. The relationships between the physicochemical properties of the tobacco leaves after fermentation and the microbial communities were examined through comparative analyses using Mantel test association network heatmaps, correlation heatmaps, and microbial network analyses. Understanding the complex interactions between microbial communities and the physicochemical properties of tobacco leaves enables better optimization of the fermentation process and aids in selecting suitable bacterial combinations, ultimately improving the quality of the tobacco leaves. This study provides scientific evidence for the contribution of pectin-degrading bacteria to the quality of cigar fermentation.

## 2 Materials and methods

### 2.1 Isolation and identification of pectin-degrading bacteria

Using the dilution plate method, healthy cigar tobacco leaf samples were ground with a mortar. 1 g of the ground sample was added to a test tube, and sterile water was added until the total volume reached the 10 mL mark. The tube was then sealed and shaken vigorously for 30 min. The supernatant was serially diluted to 10^4^ and 10^5^ fold, and 100 μL of the diluted solution was evenly spread onto NA medium. The plates were incubated at 28°C in a constant temperature incubator for 3 days. Colonies with distinct morphologies were isolated and purified on NA medium. The purified strains were then inoculated onto pectinase screening medium, and after 48 h, the formation of clear zones was observed. The hydrolysis capacity value (HC value) was then calculated using the following formula (Harnvoravongchai et al., 2020):


$$HC=\frac{d_{1}}{d_{0}}$$

In the formula: d_1_ represents the diameter of the clear zone (including the colony) in millimeters (mm), and d_0_ represents the diameter of the colony in millimeters (mm).

The 16S rDNA sequence is a crucial marker for species identification. The 16S rDNA gene of the strain was amplified using universal primers 27F-1492R via PCR. The forward primer used was 27F (sequence: TACGGYTACCTTGTTACGACTT), and the reverse primer was 1492R (sequence: AGAGTTTGATCMTGGCTCAG). The amplified product was approximately 1.5 K in length, and the PCR product was sequenced by Shanghai Rongxu Biotechnology Co., Ltd. The sequencing results were submitted to the NCBI (National Center for Biotechnology Information) database^1^ for nucleic acid sequence comparison using the BLAST program. A phylogenetic tree was constructed using the neighbor-joining method in MEGA11 software.

### 2.2 Pectin-degrading bacterial enzyme activity assay

The enzyme activity of the strains was determined using the DNS pectinase assay kit from Nanjing Jiancheng Bioengineering Institute. Pectin-degrading bacteria were inoculated into a medium containing pectin as the sole carbon source to induce the production of pectinase. After a period of cultivation, the bacterial culture was collected and centrifuged, and the supernatant was used as the enzyme solution. A 50 mL aliquot of enzyme solution was taken, with 50 mL of distilled water used as the blank control. Pectin substrate solution was added, and the enzymatic reaction was carried out at 35°C and pH 5.5. After a set period, DNS reagent was added to terminate the reaction, and the mixture was placed in a boiling water bath for 5 min. The absorbance was measured using a spectrophotometer at a wavelength of 540 nm, and the OD value of each tube was recorded. The enzyme activity was then calculated by inputting the measured data into the corresponding formula.


$$\begin{array}{c} CL\;\mathrm{activity}\;\left(U/\mathrm{mL}\right)=\frac{\left(A_{\mathrm{test}}-A_{\mathrm{control}}\right)}{A_{\mathrm{standard}}-A_{\mathrm{blank}}}\times C_{\mathrm{standard}}\\ \times V_{\mathrm{total}}\times \left(\frac{V_{\mathrm{sample}}}{V_{\mathrm{extraction}}}\times W\right)/T \end{array}$$

### 2.3 Cigar fermentation experiment design

**Figure 1:**
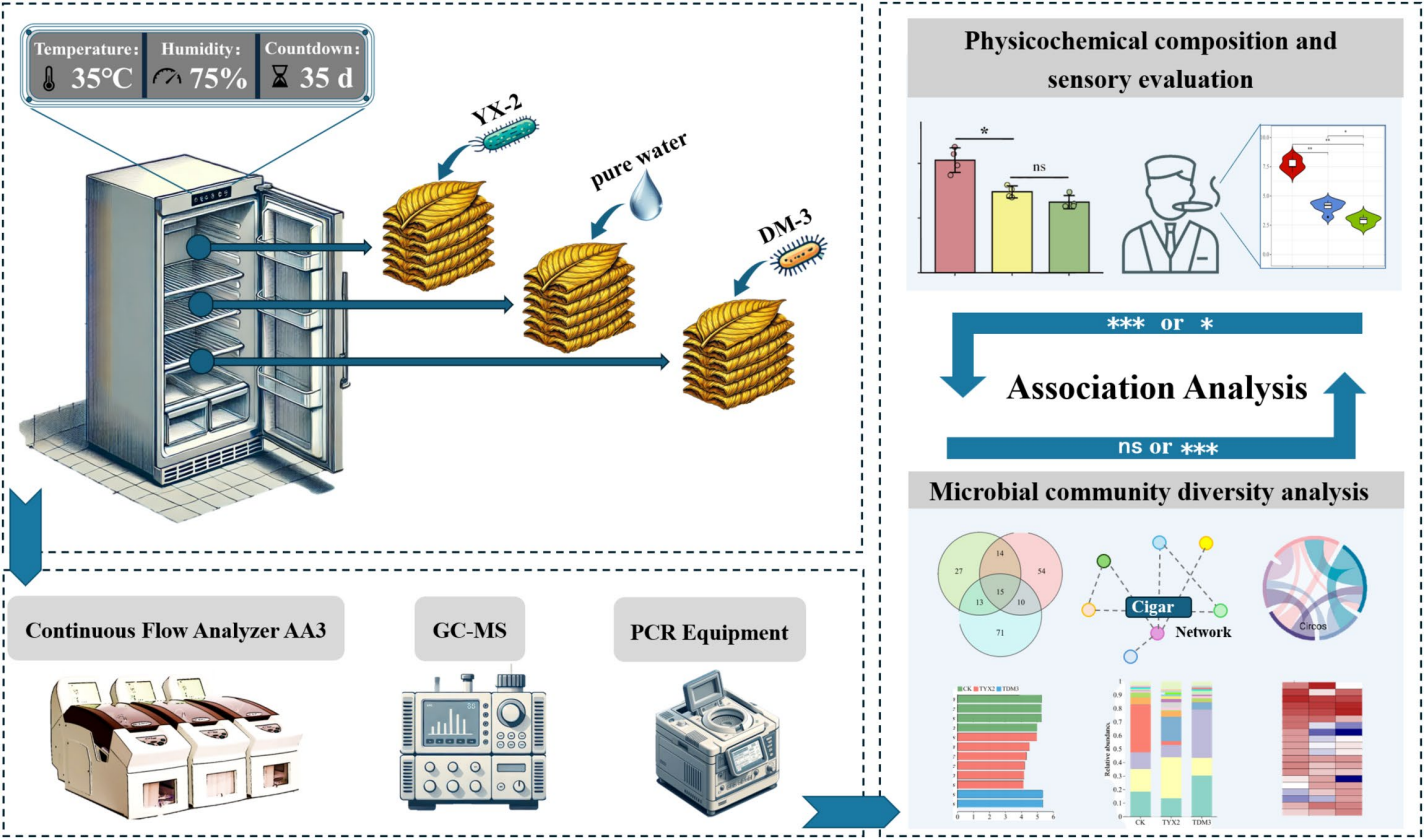
Experimental design.

Uniformly sized cigar tobacco leaves without mechanical damage or infection were selected for fermentation in an incubator. The fermentation conditions in this study simulated the actual industrial fermentation conditions of the Modu Cigar Tobacco Drying and Fermentation Management Workshop in Ede, Mosha Town, Xinping County, Yuxi City, Yunnan Province, China. To assess the impact of pectin-degrading bacteria on the quality of cigar fermentation, the two most potent strains were selected and each was inoculated into 150 mL of NB medium. The cultures were incubated at 180 r/min and 28°C for 48 h. After incubation, the seed cultures were centrifuged, and the resulting cell pellets were resuspended in 150 mL of distilled water to prepare the bacterial fermentation solutions. Three treatment groups were designed, with specific inoculants as follows: pure water, 1 × 10^8^ CFU/mL YX-2 fermentation broth, and 1 × 10^8^ CFU/mL DM-3 fermentation broth. These groups were: the CK group (fermented with pure water only), the TYX2 group (fermented with YX-2 fermentation broth only), and the TDM3 group (fermented with DM-3 fermentation broth only). Each treatment had 5 replicates. The ratio of fermentation broth to tobacco leaf mass was set at 1:50. The detailed experimental design is depicted in Figure 1. After fermentation, tobacco leaf samples were collected to assess relevant physicochemical properties and analyze microbial community diversity.

### 2.4 Analysis of physicochemical composition and sensory evaluation of tobacco leaf

Samples for analysis were uniformly collected from the center of the cigar tobacco leaves in each treatment group. The samples were ground into a fine powder using a pestle in liquid nitrogen and subsequently stored in an ultra-low temperature freezer at −80°C. The physicochemical composition of the tobacco leaves, including total nitrogen, pectin, total sugars, reducing sugars, and total alkaloids, was then measured. Refer to Table 1 for the standards on which the test methods are based (Fu et al., 2024; Ren et al., 2023).

**Table 1:** Methods and equipment for testing physicochemical composition of cigar.

Physicochemical composition	Detection method follows the standard
Total nitrogen (TN)	Tobacco and tobacco products-Determination of total nitrogenContinuous flow method (YC/T 161–2002)
Pectin	Tobacco and tobacco products—Determination of pectin—Ion chromatographic method (YC/T 346–2010)
Total alkaloid (TA)	Tobacco and tobacco products-Determination of total alkaloids-Continuous flow (potassium thiocyanate) method (YC/T 468–2013)
Total sugar (TS)	Determination of water-soluble sugars in tobacco and tobacco products by continuous flow method (YC/T 159–2019)
Reducing sugar (RS)	Determination of reducing sugar in sugar beet root (NY/T 1751–2009)

A gas chromatography–mass spectrometry (GC–MS) system (7890A/5975C, Agilent, United States) was used to analyze aroma components. The tobacco leaves were dried, ground, and sieved, and 20.00 grams of the sample was weighed and mixed with 350 mL of distilled water and 1 mL of 20 μg/mL internal standard solution (n-heptadecane) in a 1-liter round-bottom flask. Separately, 60 mL of dichloromethane was added to a 100 mL flat-bottom flask as the extraction solvent. The round-bottom flask was heated to boiling using a heating mantle, while the extraction flask was heated in a 60°C water bath, and the extraction was conducted for 3 h. The dichloromethane extract was then dried overnight with 10 grams of anhydrous sodium sulfate, concentrated to 1 mL, and transferred to a chromatography vial for GC–MS analysis. The aroma compounds were classified according to their precursor substances into chlorophyll degradation products, carotenoid degradation products, Maillard reaction products, and cembranoid degradation products (Rodriguez-Bustamante et al., 2005).

After fermentation, the samples were rolled into finished cigars and randomly labeled. Five sensory evaluation experts were organized to assess green bitterness in the cigars, following the GB 15269.4–2011 national standard, *Cigars - Part 4: Technical Requirements for Sensory Evaluation* (Weng et al., 2024). The total score for this evaluation was 10 points, with 0 < b ≤ 2.5 indicating “No green bitterness,” 2.5 < b ≤ 5.0 indicating “Mild green bitterness,” 5.0 < b ≤ 7.5 indicating “Moderate green bitterness,” and 7.5 < b ≤ 10 indicating “Strong green bitterness.

### 2.5 Interactive analysis of microbial diversity using the QIIME2 pipeline

First, genomic DNA was extracted and verified using 1% agarose gel electrophoresis. PCR amplification was performed with specific barcoded primers, utilizing TransStart Fastpfu DNA Polymerase and an ABI GeneAmp® 9,700 PCR system. To ensure the generation of adequately concentrated PCR products, low cycle numbers were employed, with consistent cycle numbers maintained across all samples. All samples were processed under standard experimental conditions, with five replicates per sample. The PCR products from the same sample were pooled and analyzed using 2% agarose gel electrophoresis. The amplified products were excised from the gel using the AxyPrep DNA Gel Extraction Kit, eluted with Tris–HCl, and verified by a second 2% agarose gel electrophoresis. Based on the preliminary quantification results from the gel electrophoresis, the PCR products were quantified using the QuantiFluor™-ST Blue Fluorescence Quantification System and subsequently pooled according to the sequencing requirements for each sample. For library preparation, adapter sequences were added to the target regions via PCR. The PCR products were excised from the gel using the Gel Extraction Kit, eluted with Tris–HCl buffer, and verified by 2% agarose gel electrophoresis. The DNA fragments were then denatured with sodium hydroxide to produce single-stranded DNA, prepared using the TruSeq™ DNA Sample Prep Kit. During sequencing, the adapter sequences hybridized to complementary sequences on the chip, anchoring the DNA fragments. The DNA fragments served as templates for PCR synthesis of the target sequences. Through denaturation and annealing, “bridges” were formed, followed by PCR amplification to generate DNA clusters, which were then linearized into single strands. Modified DNA polymerase and four types of fluorescently labeled dNTPs were added, with only one nucleotide being incorporated per cycle. A laser was used to scan the surface of the reaction plate, detecting the type of nucleotide added at each cycle. The “fluorescent groups” and “terminating groups” were chemically cleaved, restoring the 3′ end for continued nucleotide incorporation. Finally, the fluorescent signals collected at each cycle were analyzed to determine the sequence of the template DNA fragments.

The hypervariable V3-V4 regions of the bacterial 16S rRNA gene were amplified using the primer pair 799F (5′-AACMGGATTAGATACCCKG-3′) and 1193R (5′-ACGTCATCCCCACCTTCC-3′). The hypervariable ITS1F-ITS2R regions of the fungal gene were amplified using the primer pair ITS1F (5′-CTTGGTCATTTAGAGGAAGTAA-3′) and ITS12R (5′-GCTGCGTTCTTCATCGATGC-3′). After obtaining the paired-end reads during sequencing, the samples were demultiplexed, and the paired-end reads underwent quality control and filtering. The reads were then merged based on their overlap, resulting in optimized, quality-controlled data. The optimized data were subsequently processed using sequence denoising methods such as DADA2 or Deblur to obtain Amplicon Sequence Variant (ASV) representative sequences and their corresponding abundance information. Based on this information, a series of statistical and visualization analyses were conducted, including species taxonomy analysis, community diversity analysis, species differential analysis, correlation analysis, phylogenetic analysis, and functional prediction analysis.

### 2.6 Statistical analysis

Statistical analysis of the HC values of the strains, physicochemical properties of the tobacco leaves, and sequencing data was performed using Excel 2019 and SPSS 26.0 software. One-way ANOVA was used to assess differences in physicochemical properties, microbial diversity, and community composition of the tobacco leaves across different treatments. Pearson correlation analysis was employed to investigate potential correlations between microbial communities and the physicochemical properties of the tobacco leaves. Alpha diversity, based on the Sobs, Shannon, and Simpson indices, was utilized to examine species diversity differences between treatments. Community analysis calculations and visualizations were conducted using R software^2^ along with the packages “vegan,” “phyloseq,” “DESeq2,” and “picante.”

All data are presented as “mean ± standard error,” and graphs were generated using Excel 2019, GraphPad Prism 8.0, and Matlab 2019 software.

## 3 Results

### 3.1 Isolation and identification of pectin-degrading bacteria

**Figure 2:**
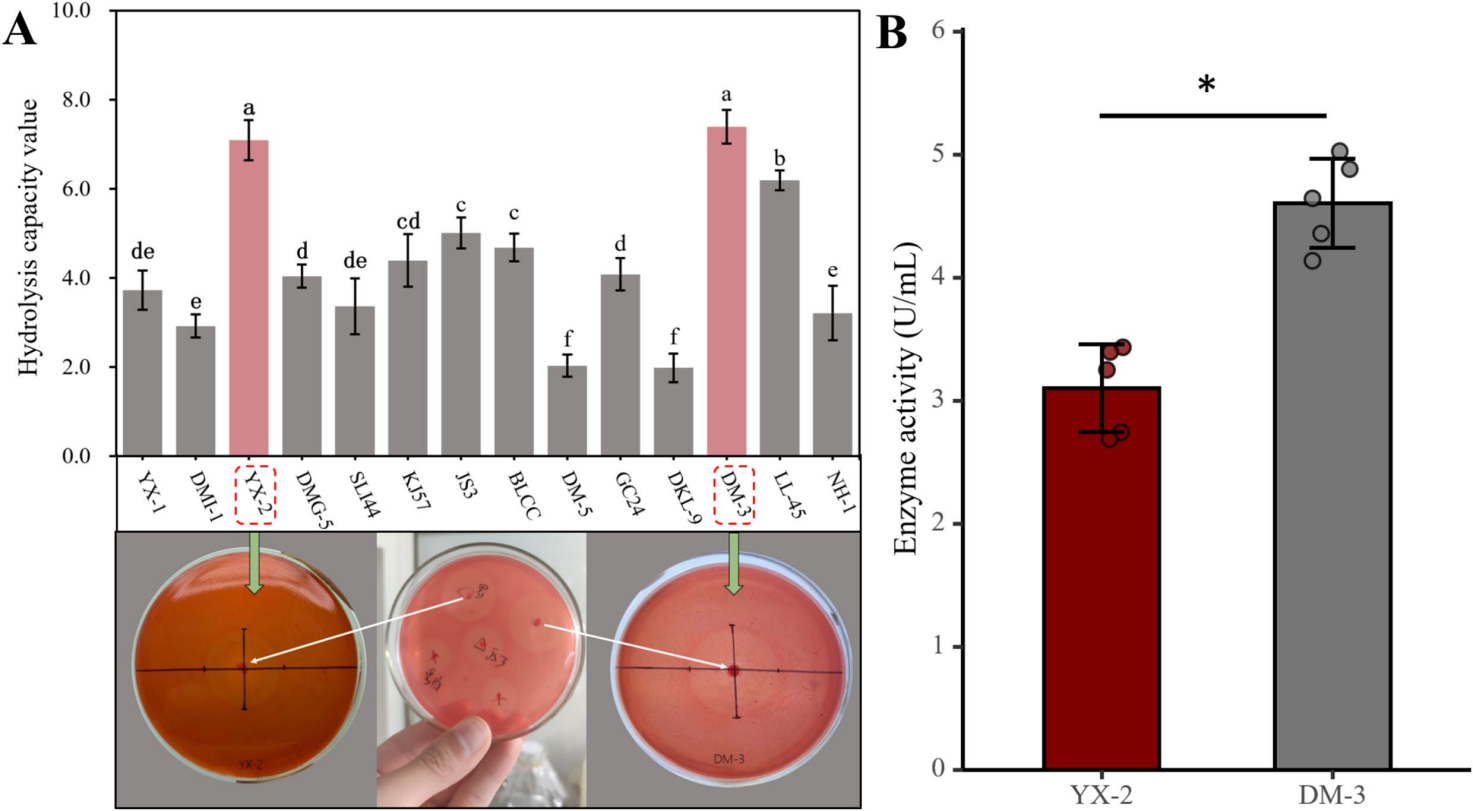
Pectinolytic strain screening results. **(A)** Shows the HC values of 14 pectinolytic strains, and **(B)** illustrates the pectinase activity of the two strongest pectinolytic strains.

The HC value is a method used to quantitatively analyze the degradation ability of microorganisms in media containing specific substrates, such as pectin. By measuring the diameter of the clear zone around the microbial colony, the enzyme activity of the strain can be assessed, enabling the rapid screening of strains with pectin-degrading capabilities. In this study, 14 pectin-degrading bacterial strains were isolated, and their degradation effects are shown in Figure 2. Among these, strains YX-2 and DM-3, isolated from ‘Yunxue No. 1’ tobacco leaves collected in Gasa Town, Xinping County, Yuxi City, Yunnan Province, formed the largest clear zones on the pectin-containing medium, with HC values significantly higher than those of other strains, indicating the strongest pectin-degrading ability. In summary, during cigar fermentation, strains YX-2 and DM-3 demonstrate considerable potential for reducing the green bitterness caused by high pectin content in tobacco leaves. Therefore, these strains were selected for further experiments in this study.

**Figure 3:**
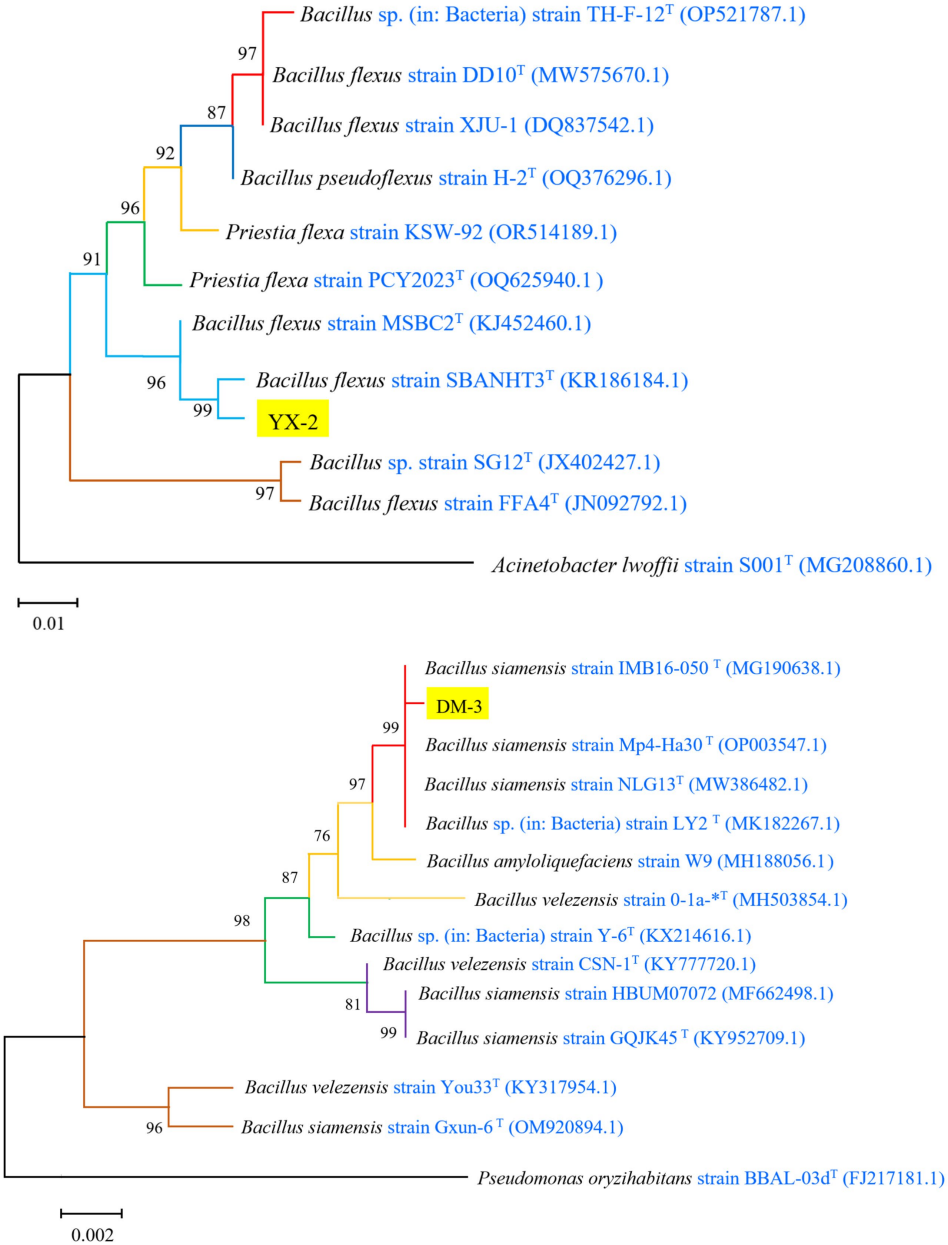
Phylogenetic tree of strains YX-2 and DM-3 constructed using the neighbor-joining method.

The 16S rDNA gene sequence of strain YX-2 was analyzed using BLAST on the NCBI platform, and reference sequences with high similarity were downloaded to construct a phylogenetic tree using MEGA11 software, as shown in Figure 3. Strain YX-2 clustered with *B. flexus* in the GenBank database, exhibiting 99.96% sequence homology with *Bacillus flexus* strain SBANHT3, indicating a highly reliable taxonomic relationship, and was preliminarily identified as *B. flexus*. Meanwhile, strain DM-3 was positioned on a branch of the phylogenetic tree near *B. siamensis*, clustering with *B. siamensis* in the GenBank database. The branches between DM-3 and its closest relatives, *Bacillus siamensis* strain IMB16-050 and *Bacillus siamensis* strain Mp4-Ha30, showed more than 99% homology, indicating that DM-3 has a high genetic similarity to these strains and was preliminarily identified as *B. siamensis*.

### 3.2 Analysis of differences in physicochemical composition among different treatment groups

**Figure 4:**
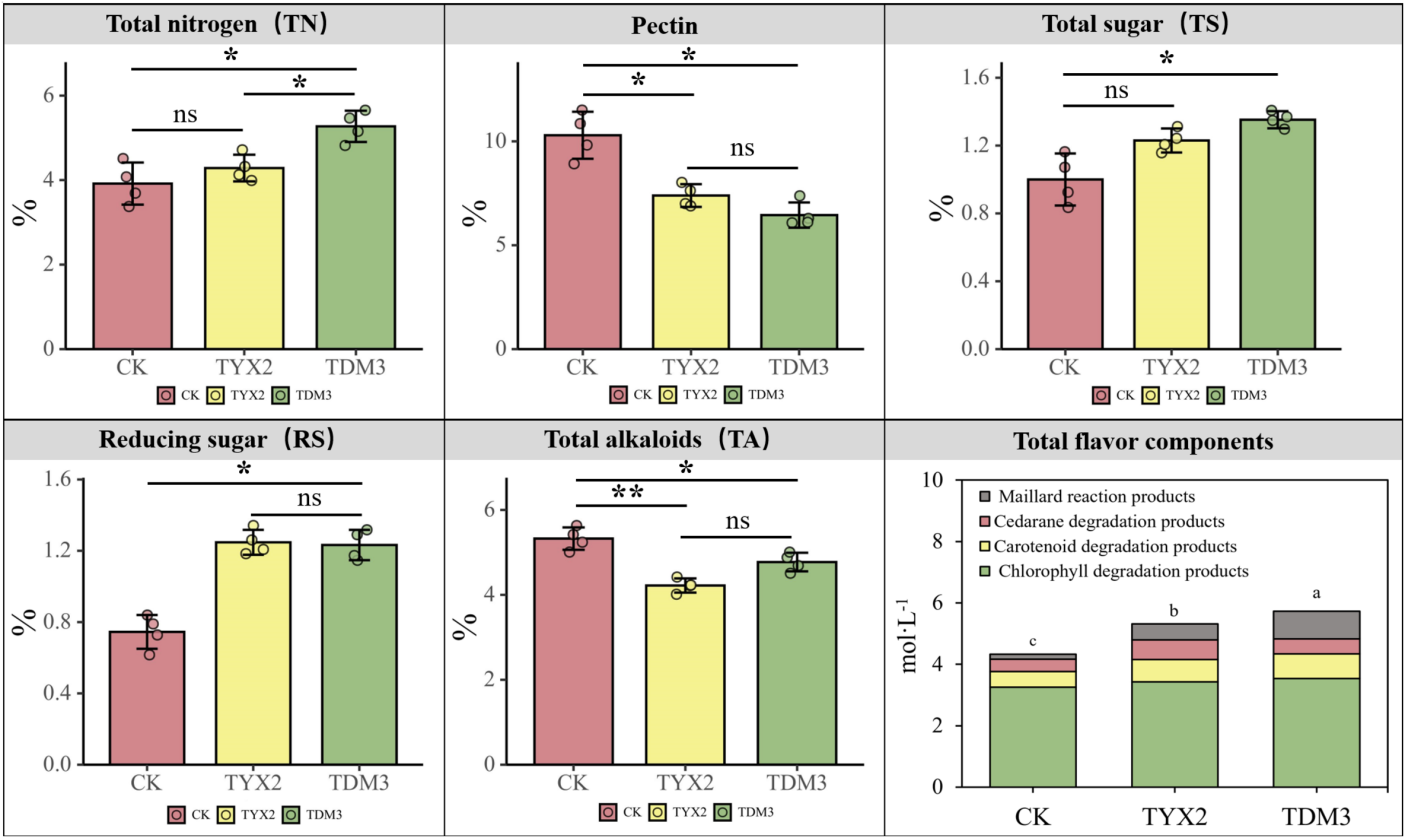
Differences in the contents of total nitrogen (TN), pectin, total sugars (TS), reducing sugars (RS), total alkaloids (TA), and total flavor components among the three treatment groups.

This study aimed to investigate the effects of two pectin-degrading bacteria on the physicochemical properties of tobacco leaves. After 35 days of fermentation, levels of total nitrogen (TN), pectin, total sugars (TS), reducing sugars (RS), total alkaloids (TA), and total flavor components were analyzed in the cigar tobacco leaves (Figure 4). In terms of total nitrogen content, both strains significantly increased levels compared to the CK group, with tobacco leaves fermented with strain DM-3 showing a total nitrogen content of approximately 5.27%, significantly higher than those in the CK and TYX2 groups. The total nitrogen content in the YX-2 strain also increased (to 4.28%) but was not as significant as in the DM-3 strain. Regarding pectin content, the addition of strains YX-2 and DM-3 significantly reduced the pectin content in the tobacco leaves to approximately 7.39 and 6.45%, respectively, which was significantly lower than the CK group’s 10.29%. This indicates that both strains YX-2 and DM-3 possess strong pectin-degrading abilities. In terms of total sugars and reducing sugars, both strains YX-2 and DM-3 significantly increased levels of total and reducing sugars in the tobacco leaves, significantly higher than those in the CK group. This suggests that these strains not only degrade pectin but also promote the hydrolysis of polysaccharides and the accumulation of sugars, enhancing the flavor of the tobacco leaves. In terms of total alkaloid content, tobacco leaves fermented with strains DM-3 and YX-2 showed significantly lower total alkaloid content compared to the CK group’s 5.32%, with strain YX-2 contributing more significantly to the reduction in alkaloid content. Regarding total flavor components, the addition of strains YX-2 and DM-3 significantly increased the levels of total flavor components in the tobacco leaves, reaching approximately 5.32 mol/L and 5.72 mol/L, respectively, significantly higher than the CK group’s 4.33 mol/L. Strain DM-3, in particular, showed more pronounced effects, especially in the production of Maillard reaction products and chlorophyll degradation products, which are important contributors to the enhancement of tobacco leaf aroma quality.

**Figure 5:**
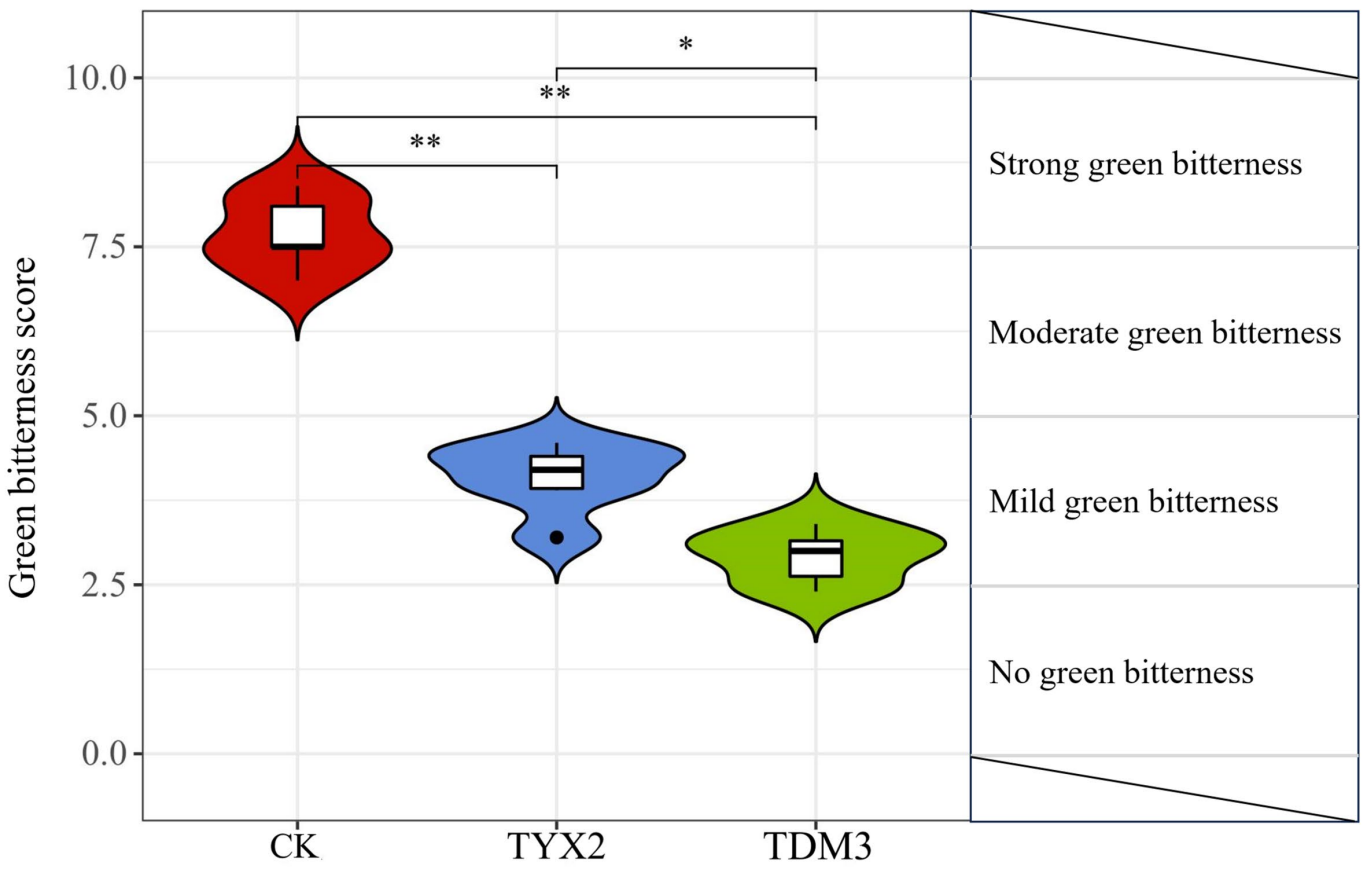
Green bitterness scores for the three treatment groups. The total score for this evaluation was 10 points, with 0 < b ≤ 2.5 indicating “No green bitterness,” 2.5 < b ≤ 5.0 indicating “Mild green bitterness,” 5.0 < b ≤ 7.5 indicating “Moderate green bitterness,” and 7.5 < b ≤ 10 indicating “Strong green bitterness.

High pectin content is a key factor contributing to the green bitterness experienced during cigar smoking. In this study, five sensory evaluation experts were invited to score the green bitterness of the three treatments (Figure 5). The results showed that after 35 days of fermentation, the CK group had the highest green bitterness score, around 7.5, indicating strong green bitterness. The TYX2 group had a significantly lower score of 4.8, indicating mild green bitterness. The TDM3 group had the lowest score, at 3.5, clearly falling into the mild green bitterness category, with a significant reduction compared to the TYX2 group. When combined with the earlier analysis of physicochemical composition, the addition of strains YX-2 and DM-3 significantly reduced the pectin content in the tobacco leaves, with both treatments showing levels significantly lower than the CK group. The reduction in pectin content directly correlates with the decrease in green bitterness, and the sensory evaluation results support this finding. Therefore, during the fermentation process of cigars, strains DM-3 and YX-2 can effectively reduce the green bitterness generated during smoking by degrading pectin, thereby improving the overall quality of the tobacco leaves.

### 3.3 Analysis of differences in tobacco leaf microbial community composition after the addition of pectin-degrading bacteria

**Figure 6:**
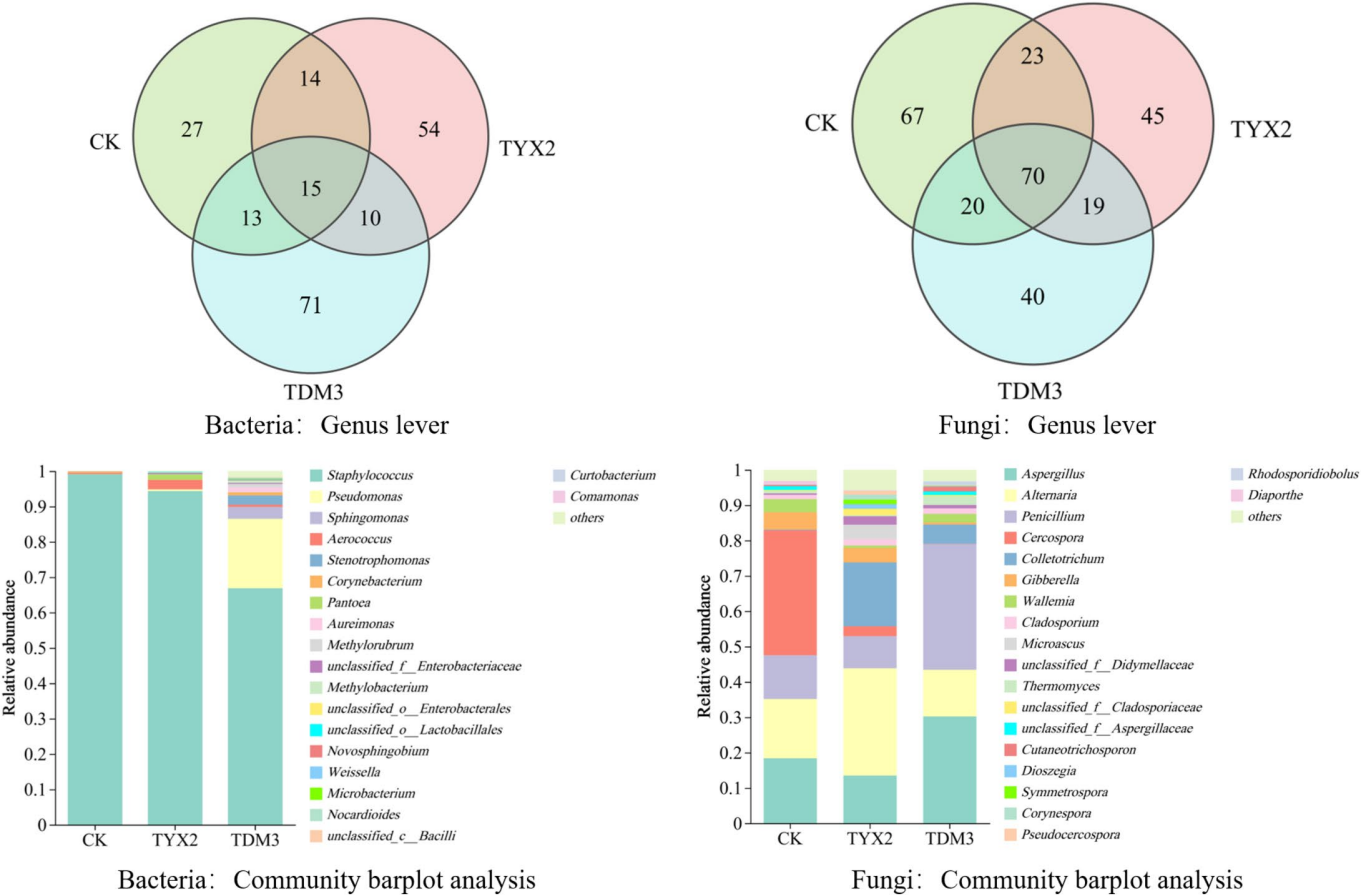
Microbial community composition of tobacco leaves after fermentation with pectin-degrading bacteria.

The Venn diagram illustrates the bacterial and fungal genus-level counts across the three treatment groups (Figure 6). The results show that the TYX2 group had 54 unique bacterial genera, and the TDM3 group had 71 unique bacterial genera, both higher than in the CK group. There were 15 common bacterial genera among the groups, indicating their general adaptability under different treatment conditions. At the fungal genus level, the CK group had 67 unique fungal genera, and 23 fungal genera were shared with both the TYX2 and TDM3 groups. After the addition of pectin-degrading bacterium YX-2 for fermentation, 45 unique fungal genera were observed, while the addition of pectin-degrading bacterium DM-3 resulted in 40 unique fungal genera. Twenty fungal genera were shared between the CK group and the other treatment groups, and 70 fungal genera were present in all three groups. These results indicate that the addition of pectin-degrading bacteria significantly influenced the fungal community structure. Notably, the TDM3 group exhibited higher fungal diversity compared to the CK and TYX2 groups. Comparing the relative abundance of fungal and bacterial genera among the three treatment groups, it is clear that the addition of pectin-degrading bacteria significantly alters the community composition. At the bacterial genus level, the CK group was predominantly composed of *Staphylococcus* and *Pseudomonas*, with *Staphylococcus* showing an extremely high abundance, exceeding 98%. In contrast, the TYX2 and TDM3 groups showed a marked reduction in *Staphylococcus* abundance, particularly in the TDM3 group, indicating that pectin-degrading bacteria can significantly decrease *Staphylococcus* abundance. The TDM3 group also exhibited greater diversity, with the relative abundance of *Pseudomonas* significantly higher than in the other treatments. Other notable bacterial genera in this group included *Methylorubrum*, *Methylobacterium*, and various unclassified *Enterobacteriaceae* and *Lactobacillales*. At the fungal genus level, the differences were even more pronounced. For example, the relative abundance of *Aspergillus* and *Penicillium* was highest in the TDM3 group, significantly higher than in the other two groups, making them key differential microorganisms. *Alternaria* was most abundant in the TYX2 group, with minimal differences between the other two groups. *Cercospora* also emerged as a differential microorganism among the three groups, with the highest relative abundance in the CK group, which was significantly reduced in the pectin-degrading bacteria-treated tobacco leaves.

### 3.4 Microbial diversity index analysis

**Figure 7:**
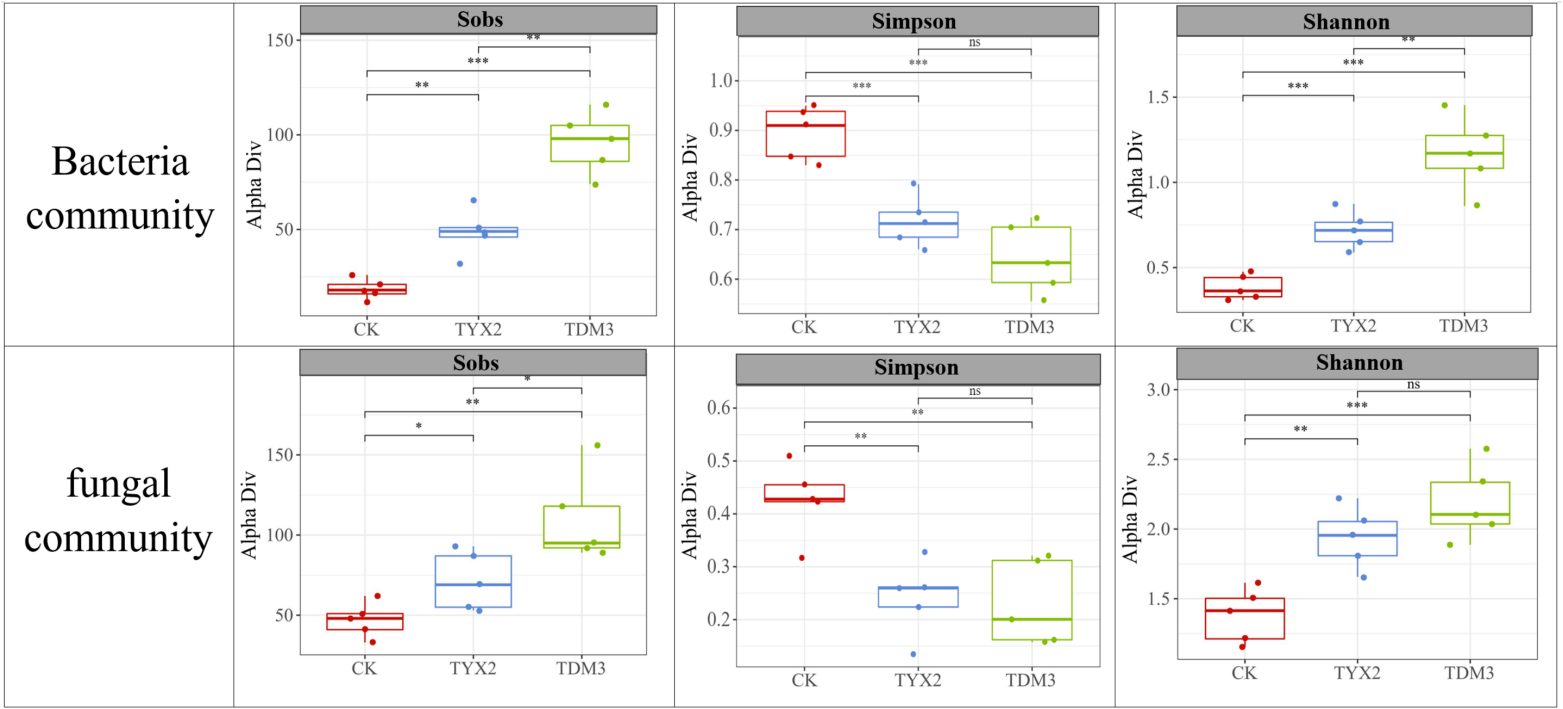
Differences in microbial diversity among the three tobacco leaf treatments.

Figure 7 illustrates the diversity indices of bacterial and fungal communities across different treatment groups. The Sobs index shows that the CK group had a relatively low number of species, while the TYX2 group exhibited a significant increase, and the TDM3 group had the highest number of species, significantly exceeding both the CK and TYX2 groups. This indicates that the addition of pectin-degrading bacteria, particularly DM-3, significantly enhances bacterial species richness, leading to a more diverse microbial community. The Sobs index for fungal communities followed a similar pattern. The Simpson index, where a lower value indicates higher community diversity, revealed that the CK group had a relatively high Simpson index, indicating lower diversity. In contrast, the TYX2 group showed a significant increase in diversity, with the Simpson index decreasing to 0.71. The TDM3 group exhibited the highest diversity, with the Simpson index further decreasing to approximately 0.68. This suggests that the inclusion of DM-3 significantly improved community diversity. The Simpson index for fungal communities showed a similar trend, indicating that pectin-degrading bacteria also positively impact fungal community diversity. The Shannon index results further support these findings, showing that the CK group had the lowest overall diversity in both bacterial and fungal communities. The TYX2 and TDM3 groups showed significant increases in Shannon index values compared to the CK group, with TDM3 being significantly higher than TYX2. This further confirms the significant role of pectin-degrading bacteria, especially DM-3, in enhancing microbial community diversity.

### 3.5 The impact of pectin-degrading bacteria on genus-level species differences in cigar tobacco leaves

**Figure 8:**
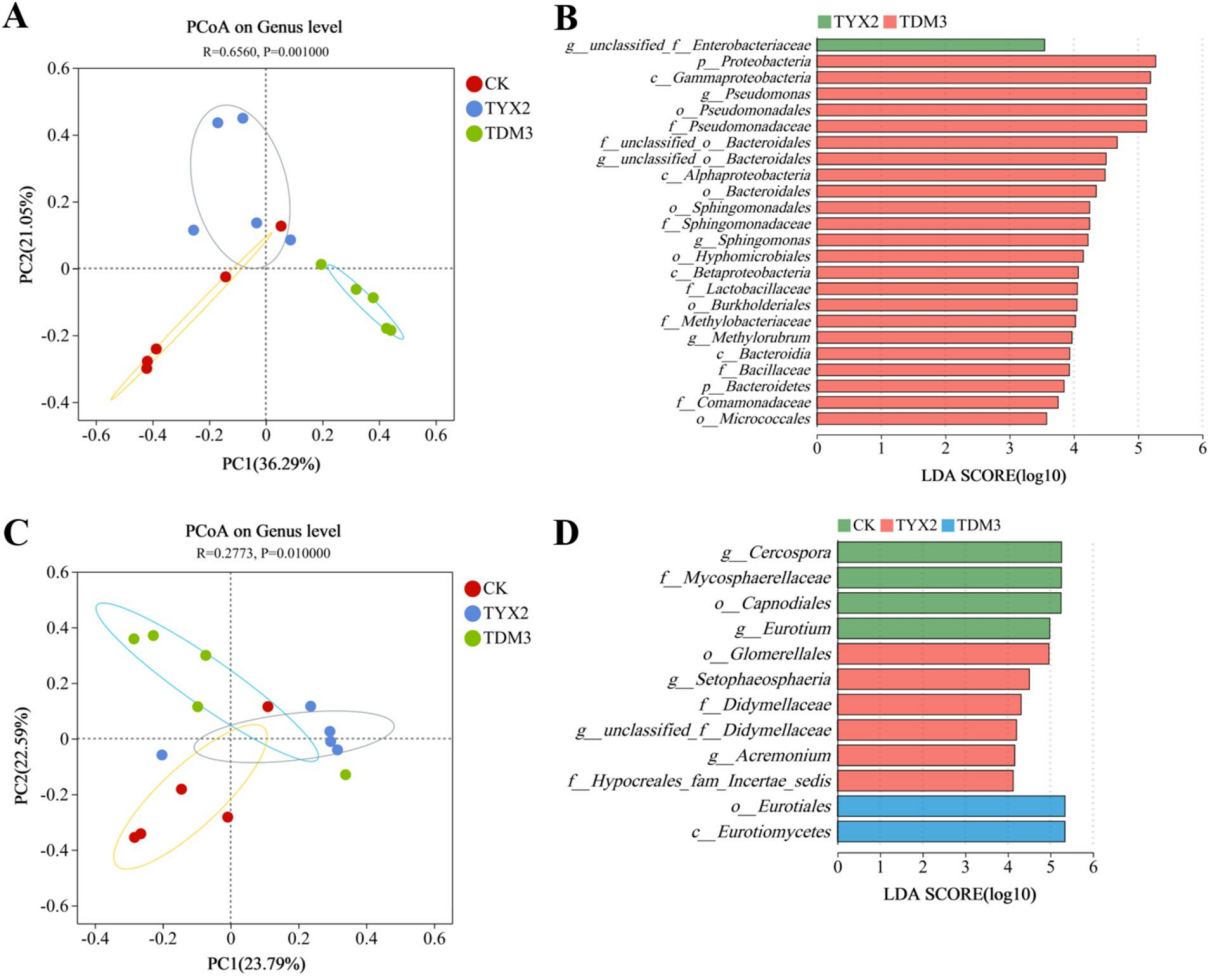
Effect of pectin-degrading bacteria fermentation on the differences in tobacco leaf microbial community composition. **(A)** PCoA of bacterial communities; **(B)** LDA effect size of bacterial communities; **(C)** PCoA of fungal communities; **(D)** LDA effect size of fungal communities. In the LDA vertical axis, lowercase letters p, c, o, f, and g represent the taxonomic levels of phylum, class, order, family, and genus, respectively.

Figure 8 shows the principal coordinates analysis (PCoA) and linear discriminant analysis (LDA) effect size of bacterial and fungal community structures during tobacco leaf fermentation. The PCoA results for bacterial communities indicate that the CK group had a relatively concentrated structure, suggesting stability. The TYX2 group displayed some dispersion, but with a clear distinction from the CK group. In contrast, the TDM3 group showed a more concentrated distribution, with significant differentiation from both the CK and TYX2 groups, indicating that strain DM-3 had a substantial impact on bacterial community structure. LEfSe analysis with an LDA threshold of 4 further revealed significant differences in specific bacterial taxa and their LDA scores. In the TYX2 group, unclassified Enterobacteriaceae were significantly enriched, suggesting their potential importance in this treatment. In the TDM3 group, Proteobacteria and the genus *Pseudomonas* were significantly enriched, with LDA scores exceeding 5, indicating that strain DM-3 promoted the enrichment of these bacteria. Regarding fungal communities, the structures in the three treatment groups also showed some dispersion, with clear distinctions between them, although the differences were less pronounced than in the bacterial communities. The LDA results indicated that in the CK group, *Eurotiales* and *Cercospora* were significantly enriched, suggesting these fungi were dominant in this group. In the TYX2 group, *Setophaeosphaeria* and *Acremonium* were significantly enriched, while in the TDM3 group, *Eurotiales* and *Eurotiomycetes* were significantly enriched. The dominant microorganisms varied across the three treatment groups. In summary, the addition of strains YX-2 and DM-3 significantly altered the microbial community structure during tobacco leaf fermentation, enhancing the enrichment of functional microorganisms. The TDM3 group, in particular, showed more pronounced effects, with these significantly enriched microorganisms likely playing crucial roles in improving pectin degradation efficiency, promoting the production of beneficial metabolites, and optimizing the fermentation environment.

**Figure 9:**
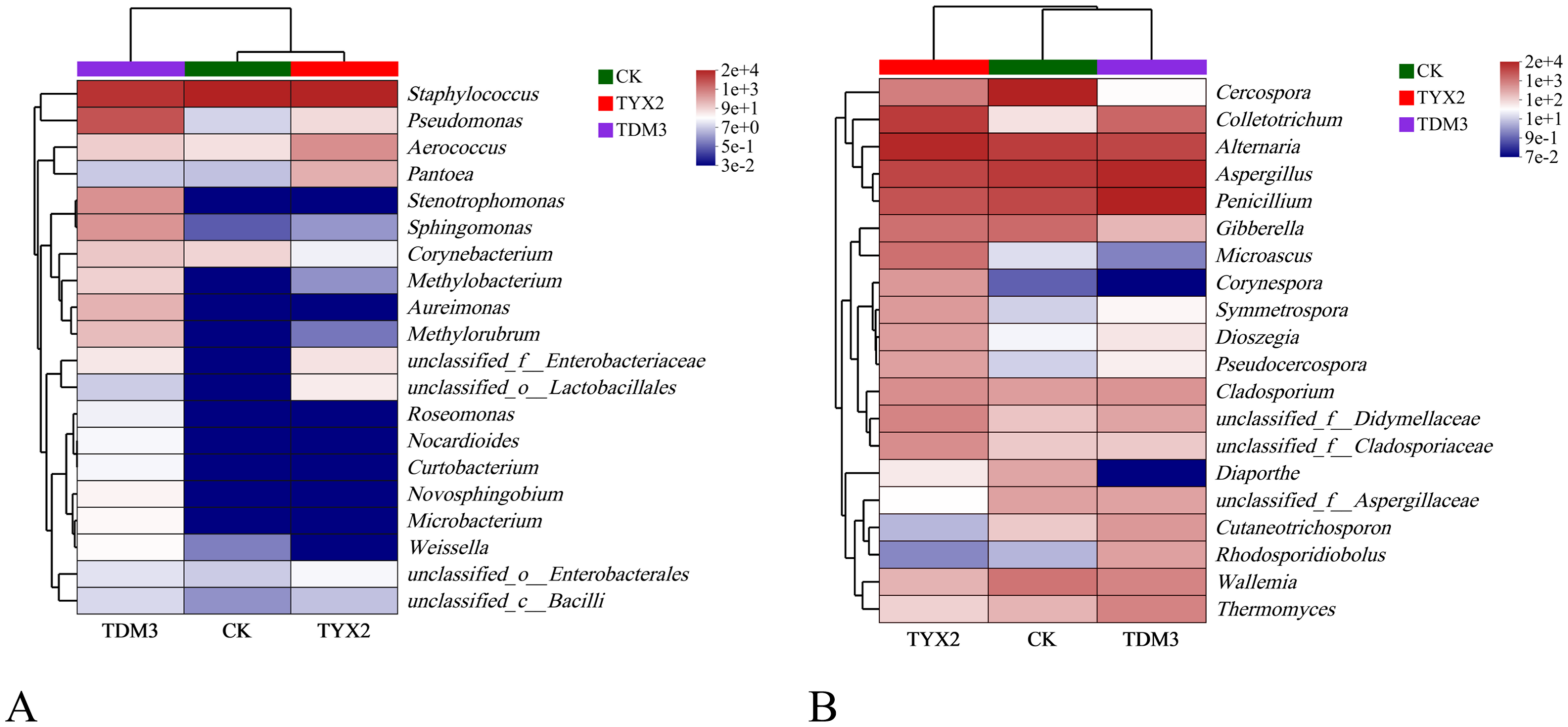
Heatmap of the microbial community in cigar tobacco leaves. In the legend, ‘e’ represents the exponent in scientific notation, indicating the magnitude of the values. **(A)** Bacterial community heatmap. **(B)** Fungal community heatmap.

As shown in the bacterial and fungal community heatmap (Figure 9), different treatment groups significantly influenced the composition and relative abundance of microbial community during fermentation. Among the bacteria, *Pseudomonas* emerged as a notably differential species, with significant differences in abundance across the three treatments: highest in the TDM3 group, followed by the TYX2 group, and lowest in the CK group. Additionally, several other bacterial genera, including *Stenotrophomonas*, *Sphingomonas*, *Methylobacterium*, and *Aureimonas*, exhibited significant differences in abundance across the three groups. Overall, the bacterial community in cigars fermented with DM-3 showed higher abundance compared to the other groups. In the fungal community, the genera *Cercospora*, *Diaporthe*, *Microascus*, and *Symmetrospora* demonstrated significant differences in abundance across the three treatments. Notably, *Diaporthe* showed extremely low abundance in the TDM3 group, moderate abundance in the TYX2 group, and high abundance in the CK group, indicating that the addition of pectin-degrading bacteria reduces the abundance of this genus within the community, making it a distinct differential species.

### 3.6 Analysis of the association between tobacco leaf physicochemical composition and microbial community

**Figure 10:**
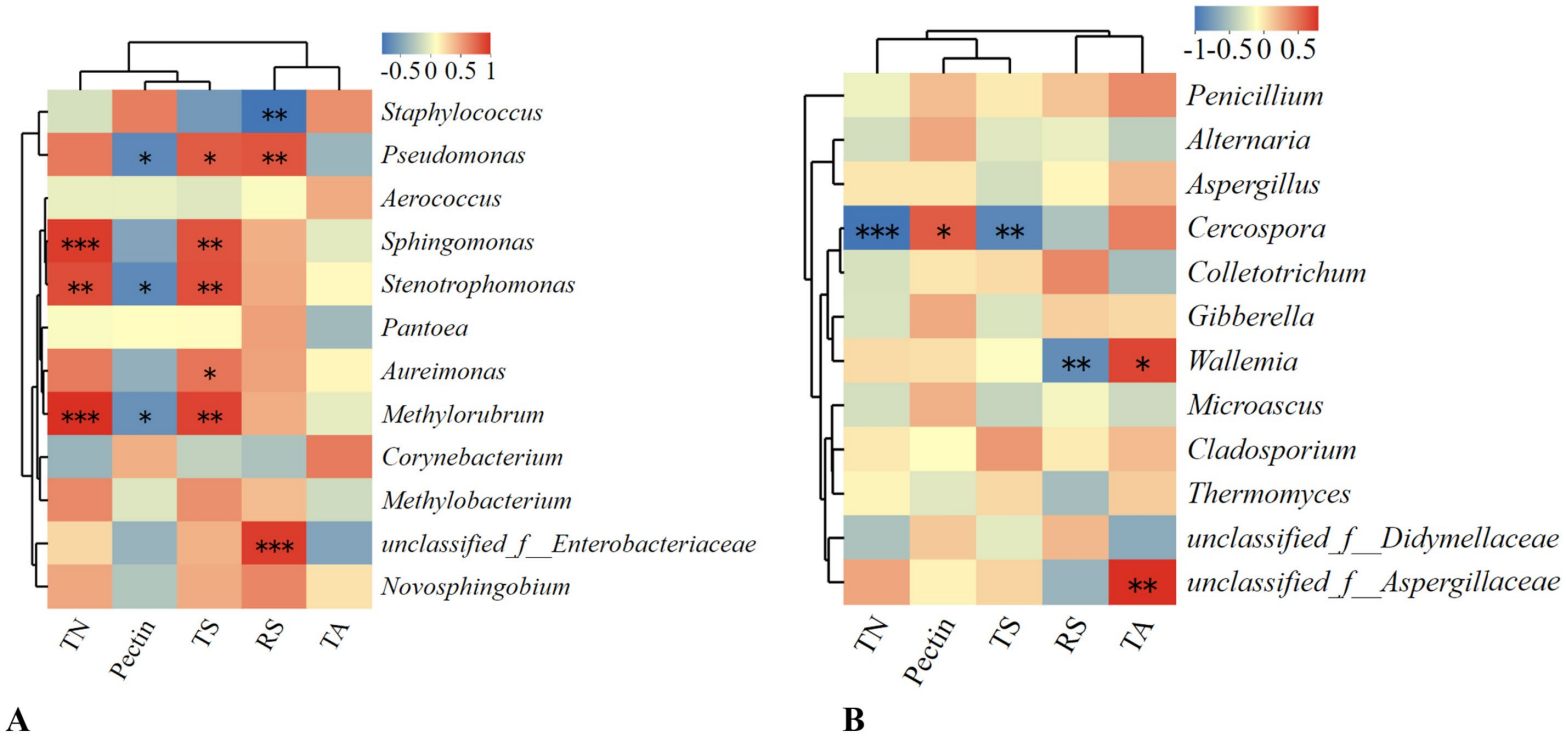
Spearman correlation heatmap between different microorganisms and the physicochemical components of tobacco leaves. **(A)** Spearman correlation heatmap of bacterial communities. **(B)** Spearman correlation heatmap of fungal communities.

Figure 10 presents the Spearman correlation heatmap between various bacterial and fungal species and the physicochemical properties of tobacco leaves, including total nitrogen (TN), pectin, total sugars (TS), reducing sugars (RS), and total alkaloids (TA). The results indicate that the abundance of *Pseudomonas*, *Stenotrophomonas*, and *Methylobacterium* is significantly negatively correlated with pectin content, suggesting that an increase in the abundance of these bacteria can lead to a reduction in pectin content. These bacteria likely play a crucial role in the degradation or indirect breakdown of pectin during tobacco leaf fermentation, contributing to a reduction in green bitterness. *Pseudomonas* also shows a significant positive correlation with both total sugars and reducing sugars, indicating that an increase in *Pseudomonas* abundance may enhance the levels of these sugars. As total sugars and reducing sugars are essential precursors of cigar aroma components, their high content can effectively enhance the flavor of cigars. Therefore, *Pseudomonas* is crucial in cigar fermentation; its high abundance not only reduces pectin content but also enhances tobacco leaf aroma to some extent. The addition of pectin-degrading bacteria during fermentation notably increases the abundance of *Pseudomonas*. On the fungal side, *Cercospora* shows a significant negative correlation with total nitrogen and total sugars, indicating that high abundance of this fungus may decrease these components, which could be detrimental to the formation of cigar aroma. These correlations suggest that specific microorganisms may significantly impact key physicochemical parameters during the fermentation of tobacco leaves.

**Figure 11:**
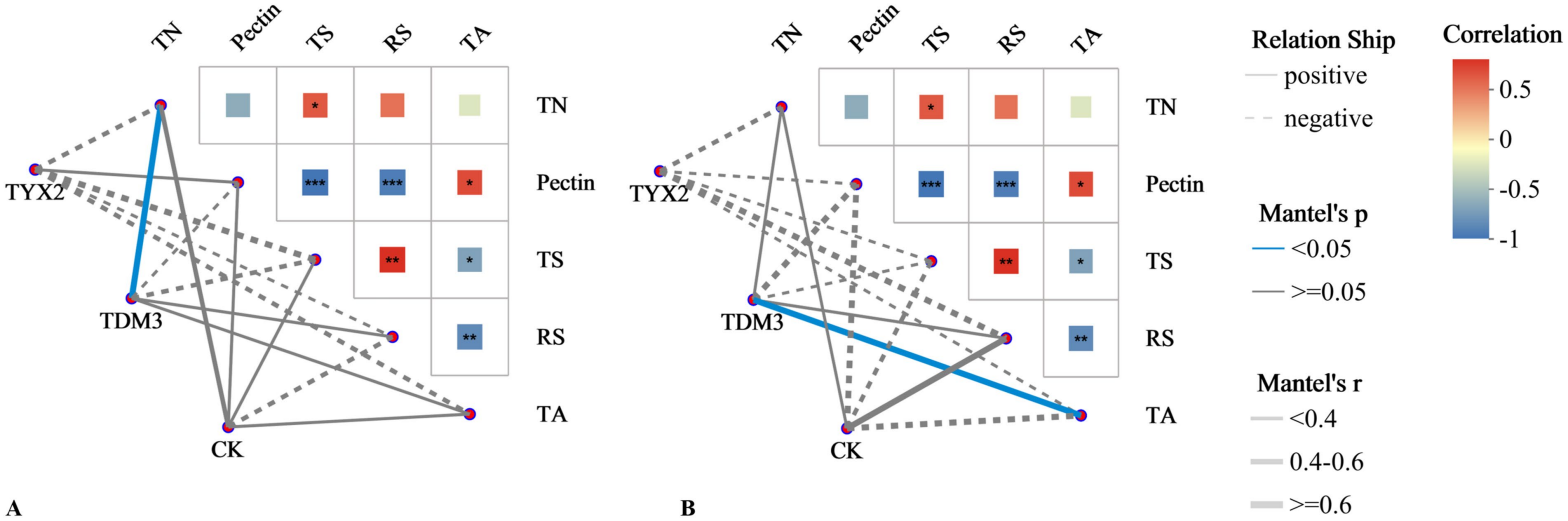
Mantel test correlation network heatmap.

To better understand the mechanisms and potential impacts of strains YX-2 and DM-3 during tobacco leaf fermentation, a Mantel test correlation network heatmap was generated (Figure 11). As shown in the figure, Mantel test analysis of bacterial communities and physicochemical properties revealed a strong and significant positive correlation between total nitrogen and total sugars and the bacterial community in the TDM3 group. This suggests that the addition of strain DM-3 contributes to the increase in total nitrogen and total sugars. Pectin showed a significant negative correlation with the bacterial community in the TDM3 group, indicating that in the TDM3 treatment, the bacterial community effectively degrades pectin. This aligns with previous results, confirming that DM-3 possesses strong pectin-degrading capabilities. Regarding fungal communities, the addition of strains YX-2 and DM-3 also significantly affected the physicochemical properties of the tobacco leaves. In the TDM3 group, pectin displayed a significant negative correlation, indicating that strain DM-3 plays a critical role in pectin degradation not only within the bacterial community but also within the fungal community.

### 3.7 Microbial network analysis

**Figure 12:**
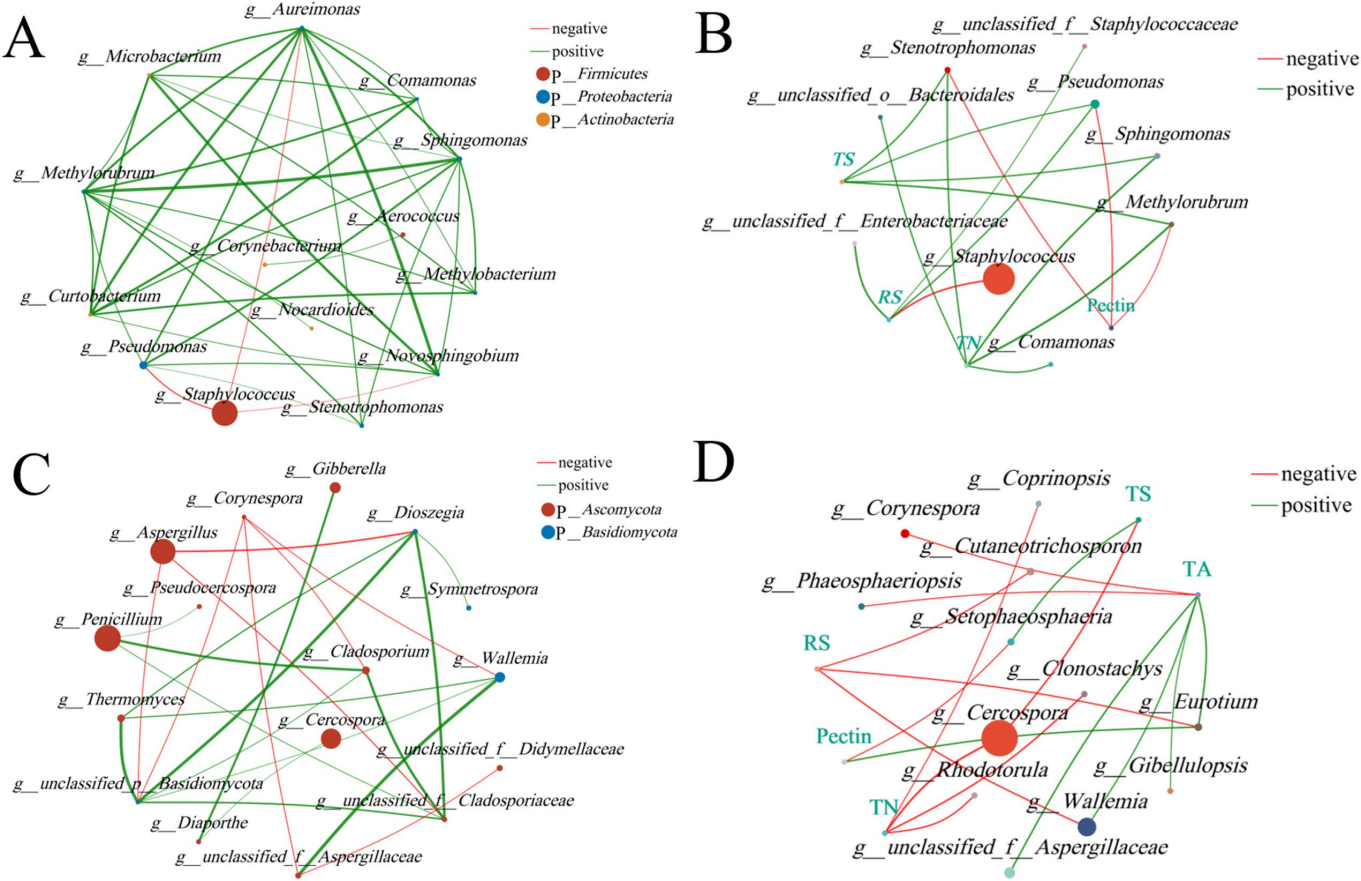
Correlation network analysis of fungal communities in tobacco leaves. **(A)** Bacterial univariate correlation network. **(B)** Bacterial bivariate correlation network. **(C)** Fungal univariate correlation network, and **(D)** Fungal bivariate correlation network. All results are presented at the genus level, with the top 20 most abundant taxa selected for both univariate and bivariate correlation networks. Correlation coefficient ≥ 0.5, *p* < 0.05.

A univariate correlation network can be used to analyze the relationships between species (Figures 12A,C), reflecting the interactions among species in tobacco leaves. *Staphylococcus* exhibited the highest abundance and displayed numerous negative correlations, indicating its significant regulatory role within the bacterial community. It showed a significant negative correlation with several bacterial genera, such as *Pseudomonas*, *Aureimonas*, and *Novosphingobium*, suggesting potential competitive interactions between these genera under certain conditions. *Methylobacterium* and *Methylorubrum* displayed many positive correlations, indicating that these bacteria might cooperate during fermentation to promote certain metabolic activities. In the fungal community, *Penicillium* and *Aspergillus* were highly abundant, with *Penicillium* showing positive correlations with three other fungi and *Aspergillus* exhibiting significant negative correlations with several fungal genera, such as *Dioszegia*, indicating potential competitive interactions among these fungi under specific conditions. *Dioszegia* and *Wallemia* demonstrated several positive correlations, suggesting that these fungi might work together during fermentation to promote pectin degradation and other metabolic activities.

The bivariate correlation network focuses on analyzing the relationships between species and physicochemical factors, helping to understand the interactions between species and the physicochemical properties of cigar tobacco leaves (Figures 12B,D). The results showed that *Pseudomonas* was positively correlated with reducing sugars (RS) and total sugars, while negatively correlated with pectin, suggesting that *Pseudomonas* plays an important role in carbohydrate metabolism and may contribute to the degradation of pectin in cigar fermentation. *Staphylococcus* was highly abundant and mainly negatively correlated with reducing sugars (RS), indicating that it may influence the accumulation of reducing sugars during fermentation through certain pathways. In the fungal community, *Cercospora*, which was highly abundant, was negatively correlated with total sugars (TS) and total nitrogen (TN), indicating that *Cercospora* may play a significant role in carbohydrate metabolism and accumulation. *Eurotium* was negatively correlated with pectin and positively correlated with reducing sugars (RS), suggesting that *Eurotium* might enhance reducing sugar content by degrading pectin during fermentation. This species appears to be important for pectin degradation, reducing green bitterness, and improving the sensory quality of tobacco leaves. Analysis of these correlation networks reveals that different treatment groups (CK, TYX2, and TDM3) significantly influence the structure of bacterial and fungal communities and their relationships with the physicochemical properties of tobacco leaves.

## 4 Discussion

### 4.1 Mechanisms underlying the enhancement of cigar quality by pectin-degrading bacteria

This study demonstrates that the addition of strains YX-2 and DM-3 significantly reduced the pectin content in cigar tobacco leaves, thereby decreasing green bitterness and improving the overall quality of the tobacco. Pectin, a key polysaccharide in tobacco leaves, produces organic acids and other byproducts during its degradation, which are major contributors to green bitterness (Zhu et al., 2015). The physicochemical analysis in this study revealed that the pectin content in tobacco leaves was significantly lower in the YX-2 and DM-3 groups compared to the CK group, with a corresponding significant reduction in green bitterness, confirming the efficacy of these strains in pectin degradation. The reduction in green bitterness is primarily due to the efficient degradation capabilities of pectin-degrading bacteria, which secrete highly active pectinases, such as pectate lyase and polygalacturonase. These enzymes rapidly break down pectin into smaller molecules, including galacturonic acid and oligosaccharides, thereby reducing pectin accumulation during fermentation and subsequently decreasing the production of bitter compounds (Wang et al., 2020; Khan et al., 2013). Additionally, galacturonic acid, a primary product of pectin degradation, can cause significant bitterness at high concentrations. By efficiently degrading pectin, these bacteria reduce the accumulation of galacturonic acid, thereby lowering green bitterness (Shrestha et al., 2021; Amin et al., 2019). Moreover, pectin degradation produces certain organic acids, aldehydes, and ketones, which may contribute to green bitterness during combustion. By effectively degrading pectin, these bacteria reduce the formation of these compounds, further decreasing the green bitterness of the cigar (Adapa et al., 2014; Bonnin et al., 2014; Wen et al., 2023). Another factor contributing to the reduced bitterness is the improvement of the fermentation environment due to the addition of pectin-degrading bacteria. The low-molecular-weight compounds, such as oligosaccharides produced during pectin degradation, provide nutrients for other beneficial microorganisms, promoting their growth and optimizing the structure of the microbial community during fermentation (Deng et al., 2003; Pascale et al., 2022; Zheng et al., 2022). This synergistic effect further improves the fermentation environment, thereby reducing green bitterness. Additionally, by lowering pectin content, the formation of certain precursors to undesirable flavors can be minimized, thus reducing the production of off-flavors during fermentation. For instance, incomplete pectin degradation can lead to the formation of 2-methylbutyric acid, which has a strong unpleasant odor and bitterness, potentially affecting the overall quality of cigars (Martin et al., 2010; Zheng et al., 2021).

In terms of total aroma components, this study found that the addition of strains YX-2 and DM-3 significantly increased the total aromatic compounds in tobacco leaves. The organic acids produced from pectin degradation, such as galacturonic acid, can be further metabolized into aromatic compounds like esters and alcohols, thereby enhancing the aromatic profile of the tobacco leaves (Hansson et al., 2002). Additionally, during fermentation, pectin-degrading bacteria can promote the formation of carotenoid degradation products and Maillard reaction products, both of which play a crucial role in the unique aroma and complex flavor of cigars. For example, carotenoid degradation products can produce fruity aroma compounds, while Maillard reaction products contribute to nutty and caramel notes (Yu et al., 2023; Weng et al., 2024; Zhang et al., 2020). The data from this study indicate that Maillard reaction products were significantly higher in the DM-3 treatment group during cigar fermentation compared to other groups. This suggests that the DM-3 strain may have a unique advantage in promoting the Maillard reaction during fermentation, thereby enhancing the aroma and flavor of the cigar. The Maillard reaction is a complex chemical process between amino acids and reducing sugars under both thermal and non-thermal conditions, resulting in a series of compounds with dark colors, aromas, and flavors. Maillard reaction products (MRPs) significantly contribute to the flavor of foods and tobacco products, including the development of caramel, nutty, and roasted aromas (Tamanna and Mahmood, 2015; Lee et al., 2010). This also supports the notion that higher levels of reducing sugars play a critical role in boosting the production of Maillard reaction products, indirectly highlighting the significant contribution of reducing sugars to the flavor profile of cigars. This finding suggests that the DM-3 strain may increase the production of reducing sugars during fermentation through its metabolic pathways or by utilizing more efficient sugar metabolism pathways or enzyme activities to convert complex carbohydrates (e.g., starch, pectin) into simple sugars, thus providing sufficient substrates for the Maillard reaction and leading to a notable increase in Maillard reaction products (Hu et al., 2023; Guízar-Amezcua et al., 2022). In addition to enhancing flavor, the addition of pectin-degrading bacteria also improved the physical properties of the tobacco leaves. Pectin degradation can alter the physical structure of the leaves, making them softer and more elastic, which enhances their rollability and burning uniformity. Softer leaves are easier to roll into shape and burn more evenly during smoking, providing a better smoking experience. By reducing the pectin content, the tissue structure of the tobacco leaves becomes more relaxed, allowing smoke to pass through more smoothly, reducing resistance, and improving the comfort of the draw (Weng et al., 2024; Gong et al., 2023). However, despite the potentially significant impact of pectin-degrading bacteria on the physical properties of tobacco leaves, such as softness and elasticity, this study did not systematically evaluate the changes in the physical structure of cigar tobacco leaves. This omission represents a notable limitation of the research. Future studies should comprehensively assess the effects of pectin-degrading bacteria on the physical properties of tobacco leaves, including a systematic analysis of softness, elasticity, density, rollability, burning performance, and microstructural changes. Such research will provide valuable scientific insights into the role of pectin-degrading bacteria in the cigar fermentation process and could further optimize cigar production processes, thereby enhancing product quality and market competitiveness.

### 4.2 Pectin-degrading bacteria influence tobacco leaf quality through the regulation of microbial community

This study systematically analyzed the diversity of bacterial and fungal communities, species differentiation, and their correlations with the physicochemical properties of tobacco leaves, aiming to explore the contribution of pectin-degrading bacteria (YX-2 and DM-3) to the cigar fermentation process and subsequent quality improvement. The results demonstrated that the pectin-degrading bacteria not only significantly altered the microbial community structure but also had a profound impact on the physicochemical properties of the tobacco leaves, ultimately enhancing the overall quality of the cigars. This was primarily reflected in significant changes in the diversity and structure of the microbial community. The study observed a notable increase in the diversity of both bacterial and fungal communities following the addition of pectin-degrading bacteria. Specifically, the TDM3 group exhibited the highest diversity and species richness, as indicated by the Sobs, Shannon, and Simpson indices, which were significantly higher than those of the CK and TYX2 groups. This result suggests that the introduction of the DM-3 strain promoted the diversification of the microbial community, potentially fostering a more competitive and functional fermentation environment through nutrient competition, metabolite secretion, and microbial interactions (Zheng et al., 2022; Jia et al., 2023). Notably, within the bacterial community, key microorganisms, including those from the phylum Proteobacteria and the genus *Pseudomonas*, were significantly enriched in the TDM3 group. These microorganisms are not only closely associated with pectin degradation but may also enhance cigar flavor by promoting carbohydrate metabolism. Studies have shown that *Pseudomonas* bacteria exhibit significant metabolic activity in various fermentation processes, which affects the physicochemical properties of the fermented products (Joshi et al., 2011; Xiao et al., 2021). For instance, in tea fermentation, *Pseudomonas* has been found to influence the chemical composition of tea, particularly by promoting the accumulation of reducing sugars and total sugars. *Pseudomonas* secretes various enzymes, including pectinases and cellulases, which break down complex polysaccharides in tea, generating simple sugars that enhance the taste and flavor of the tea (Benninghaus et al., 2021; Xia et al., 2022). *Pseudomonas* also plays a crucial role in coffee bean fermentation. In this process, *Pseudomonas* degrades complex carbohydrates in coffee beans, particularly by breaking down pectin through pectinase activity, thereby reducing the astringency of the beans (Cruz-O'Byrne et al., 2021; Avallone et al., 2001). In this study, the addition of the DM-3 strain significantly promoted the enrichment and increased the abundance of *Pseudomonas*. This finding suggests that the DM-3 strain has unique advantages in the fermentation process, either directly or indirectly enhancing the dominance of *Pseudomonas* within the microbial community. The significant enrichment of *Pseudomonas* likely acts as the primary driver of pectin degradation and carbohydrate accumulation, contributing to the enhancement of aroma and flavor complexity in tobacco leaves, which is crucial for improving the quality of cigar fermentation.

*Bacillus flexus* and *Bacillus siamensis* have been proven to possess strong pectin-degrading capabilities. Numerous studies have shown that the high efficiency of these bacterial strains in pectin degradation is attributed to their high pectinase activity (Girish, 2023). For example, Weng et al. (2024) pointed out that certain *Bacillus* species can secrete various polysaccharide-degrading enzymes, with pectinase playing a crucial role in the breakdown of plant tissues. In this study, both YX-2 and DM-3 exhibited high pectinase activity, with DM-3 showing particularly strong activity, indicating a significant advantage of these strains in pectin degradation. However, there are few studies focusing specifically on the degradation of pectin in cigar tobacco during fermentation. Research has shown that pectin degradation can improve the flavor of food or tobacco. For instance, pectin degradation not only reduces undesirable textures but also increases polysaccharide content, enhancing the sensory characteristics of the fermented product (McCann et al., 1994; Bedzo et al., 2023). As noted by Yao et al. (2022) and Guo et al. (2024), regulating microbial metabolic activity in certain fermented foods can enhance the final product’s flavor. This study demonstrated that the addition of strains YX-2 and DM-3 significantly reduced the pectin content in cigar tobacco leaves, and this reduction in pectin content was closely related to the decrease in green bitterness. After treatment with these strains, the green bitterness of the cigar tobacco was noticeably reduced, with strain DM-3 showing particularly pronounced effects. This improvement directly contributed to enhancing the flavor and sensory quality of the cigar tobacco, highlighting a major advantage of these two strains. Introducing specific strains during fermentation can effectively modify the microbial community structure and promote the growth of beneficial microbes. In this study, the microbial community structure of cigar tobacco leaves underwent significant changes after treatment with pectin-degrading bacteria YX-2 and DM-3, particularly with an increase in bacterial and fungal diversity. The impact of strain DM-3 on both bacterial and fungal communities was especially notable, significantly enriching key functional microorganisms such as *Pseudomonas*, *Staphylococcus*, *Penicillium*, *Alternaria*, and *Aspergillus*. These microbes may promote pectin degradation and the production of beneficial metabolites through synergistic interactions, thereby optimizing the fermentation environment and further enhancing the overall quality of the tobacco leaves. Zhao et al. (2024) explored the role of *A. niger* in pectin degradation during the fermentation of tobacco stems. Due to its strong pectinase activity, *A. niger* acts as the primary agent of pectin hydrolysis during fermentation. The study reported that through fermentation treatment with *A. niger*, the pectin degradation efficiency in tobacco stems exceeded 90% within 7 days. The pectinase secreted by *A. niger*, especially pectate lyase, effectively breaks down the complex polysaccharide pectin, which is particularly important in the treatment of pectin-rich biomass, such as tobacco stems. The study also mentioned that during fermentation, *A. niger* can inhibit the growth of certain competing microorganisms through its metabolic products, while promoting the proliferation of key beneficial microbes such as *Pseudomonas*. This microbial community adjustment further optimizes the fermentation environment, making the process more efficient and enhancing the quality of the final fermented product. These findings are consistent with the results of the present study. Cigar tobacco fermentation is a complex process characterized by dynamic changes in microbial populations. In the future, more attention will be paid to the survival and dynamic changes of added beneficial bacteria during the fermentation process. This will provide deeper insights into the direct relationship between beneficial microbes and the improvement of tobacco leaf quality, offering more reliable references for the development of strains in future research.

## 5 Conclusion

In conclusion, our study demonstrates that the introduction of pectin-degrading bacteria significantly altered both the microbial community structure and the physicochemical properties of cigar leaves during fermentation. Compared to the control group (CK), the treatment groups with strains YX-2 and DM-3 showed increased bacterial and fungal diversity, notably with a significant enrichment of *Pseudomonas* and other functional microorganisms. Furthermore, the application of pectin-degrading bacteria effectively reduced the pectin content in the leaves, which in turn decreased the bitterness that develops during fermentation, while significantly increasing both the total sugar and reducing sugar content, thereby enhancing the aroma quality of the cigar leaves. Species difference analysis revealed that *Pseudomonas* abundance was highest in the TDM-3 treatment group, showing a significant negative correlation with pectin content and a positive correlation with both reducing sugar and total sugar content, underscoring its critical role in cigar fermentation. Furthermore, the fungal species *Eurotium* and others were significantly enriched in the TDM-3 group, which were closely associated with pectin degradation and reducing sugar accumulation. The pectin-degrading bacteria YX-2 and DM-3 not only optimized the microbial community but also improved the physicochemical properties of the leaves, thereby effectively enhancing the overall quality of the cigars. This study provides new scientific evidence and technical support for the application of pectin-degrading bacteria in cigar fermentation, offering valuable resources for the further optimization of cigar fermentation processes in the future.

## Data Availability

The data analyzed in this study was obtained from Technical Center of Yuxi Branch, Yunnan Provincial Tobacco Company. The data in this study are subject to confidentiality agreements, and some data have not been publicly released in open datasets to protect the sensitivity of the data and the privacy of the project. Requests to access these datasets should be directed to Xueru Song, florakaloo@163.com.

## Footnotes

^1^ http://www.ncbi.nlm.nih.gov

^2^Version 4.1.1, https://www.r-project.org
