# Supplementary material for: Contribution of pectin-degrading bacteria to the quality of cigar fermentation: an analysis based on microbial communities and physicochemical components
Source: Front Microbiol. 2024 Nov 14;15:1481158. doi: 10.3389/fmicb.2024.1481158 (PMC11604125; doi:10.3389/fmicb.2024.1481158)
Supplement: Supplementary file 5 [file Table_1.DOCX]

The assembled sequence of sample YX-2：

GGCGGCTGGCTCCATAAAGGTTACCCCACCGACTTCGGGTGTTACAAACTCTCGTGGTGTGACGGGCGGTGTGTACAAGGCCCGGGAACGTATTCACCGCGGCATGCTGATCCGCGATTACTAGCGATTCCAGCTTCATGTAGGCGAGTTGCAGCCTACAATCCGAACTGAGAATGGTTTTATGGGATTGGCTTGACCTCGCGGTCTTGCAGCCCTTTGTACCATCCATTGTAGCACGTGTGTAGCCCAGGTCATAAGGGGCATGATGATTTGACGTCATCCCCACCTTCCTTCCGGTTTGTCACCGGCAGTCACCTTAGAGTGCCCAACTAAATGCTGGCAACTAAGATCAAGGGTTGCGCTCGTTGCGGGACTTAACCCAACATCTCACGACACGAGCTGACGACAACCATGCACCACCTGTCACTCTGTCCCCCGAAGGGGAACGCTCTATCTCTAGAGTTGTCAGAGGATGTCAAGACCTGGTAAGGTTCTTCGCGTTGCTTCGAATTAAACCACATGCTCCACCGCTTGTGCGGGCCCCCGTCAATTCCTTTGAGTTTCAGTCTTGCGACCGTACTCCCCAGGCGGAGTGCTTAATGCGTTAGCTGCAGCACTAAAGGGCGGAAACCCTCTAACACTTAGCACTCATCGTTTACGGCGTGGACTACCAGGGTATCTAATCCTGTTTGCTCCCCACGCTTTCGCGCCTCAGCGTCAGTTACAGACCAAAAAGCCGCCTTCGCCACTGGTGTTCCTCCACATCTCTACGCATTTCACCGCTACACGTGGAATTCCGCTTTTCTCTTCTGCACTCAAGTTCCCCAGTTTCCAATGACCCTCCACGGTTGAGCCGTGGGCTTTCACATCAGACTTAAGAAACCGCCTGCGCGCGCTTTACGCCCAATAATTCCGGATAACGCTTGCCACCTACGTATTACCGCGGCTGCTGGCACGTAGTTAGCCGTGGCTTTCTGGTTAGGTACCGTCAAGGTACAAGCAGTTACTCTTGTACTTGTTCTTCCCTAACAACAGAGTTTTACGACCCGAAAGCCTTCATCACTCACGCGGCGTTGCTCCGTCAGACTTTCGTCCATTGCGGAAGATTCCCTACTGCTGCCTCCCGTAGGAGTCTGGGCCGTGTCTCAGTCCCAGTGTGGCCGATCACCCTCTCAGGTCGGCTATGCATCGTTGCCTTGGTGAGCCGTTACCTCACCAACTAGCTAATGCACCGCGGGCCCATCTGTAAGTGATAGCCGAAACCATCTTTCAATTTTCTCTTATGCAAGAGAAAATGTTATCCGGTATTAGCTCCGGTTTCCCGGAGTTATCCCAGTCTTACAGGCAGGTTGCCCACGTGTTACTCACCCGTCCGCCGCTAACGTCATAGAAGCAAGCTTCTAATCAGTTCGCTCGACTGCATGT

The assembled sequence of sample DM-3：

TTCGGCGGCTGGCTCCATAAAGGTTACCTCACCGACTTCGGGTGTTACAAACTCTCGTGGTGTGACGGGCGGTGTGTACAAGGCCCGGGAACGTATTCACCGCGGCATGCTGATCCGCGATTACTAGCGATTCCAGCTTCACGCAGTCGAGTTGCAGACTGCGATCCGAACTGAGAACAGATTTGTGGGATTGGCTTAACCTCGCGGTTTCGCTGCCCTTTGTTCTGTCCATTGTAGCACGTGTGTAGCCCAGGTCATAAGGGGCATGATGATTTGACGTCATCCCCACCTTCCTCCGGTTTGTCACCGGCAGTCACCTTAGAGTGCCCAACTGAATGCTGGCAACTAAGATCAAGGGTTGCGCTCGTTGCGGGACTTAACCCAACATCTCACGACACGAGCTGACGACAACCATGCACCACCTGTCACTCTGCCCCCGAAGGGGACGTCCTATCTCTAGGATTGTCAGAGGATGTCAAGACCTGGTAAGGTTCTTCGCGTTGCTTCGAATTAAACCACATGCTCCACCGCTTGTGCGGGCCCCCGTCAATTCCTTTGAGTTTCAGTCTTGCGACCGTACTCCCCAGGCGGAGTGCTTAATGCGTTAGCTGCAGCACTAAGGGGCGGAAACCCCCTAACACTTAGCACTCATCGTTTACGGCGTGGACTACCAGGGTATCTAATCCTGTTCGCTCCCCACGCTTTCGCTCCTCAGCGTCAGTTACAGACCAGAGAGTCGCCTTCGCCACTGGTGTTCCTCCACATCTCTACGCATTTCACCGCTACACGTGGAATTCCACTCTCCTCTTCTGCACTCAAGTTCCCCAGTTTCCAATGACCCTCCCCGGTTGAGCCGGGGGCTTTCACATCAGACTTAAGAAACCGCCTGCGAGCCCTTTACGCCCAATAATTCCGGACAACGCTTGCCACCTACGTATTACCGCGGCTGCTGGCACGTAGTTAGCCGTGGCTTTCTGGTTAGGTACCGTCAAGGTGCCGCCCTATTTGAACGGCACTTGTTCTTCCCTAACAACAGAGCTTTACGATCCGAAAACCTTCATCACTCACGCGGCGTTGCTCCGTCAGACTTTCGTCCATTGCGGAAGATTCCCTACTGCTGCCTCCCGTAGGAGTCTGGGCCGTGTCTCAGTCCCAGTGTGGCCGATCACCCTCTCAGGTCGGCTACGCATCGTCGCCTTGGTGAGCCGTTACCTCACCAACTAGCTAATGCGCCGCGGGTCCATCTGTAAGTGGTAGCCGAAGCCACCTTTTATGTCTGAACCATGCGGTTCAAACAACCATCCGGTATTAGCCCCGGTTTCCCGGAGTTATCCCAGTCTTACAGGCAGGTTACCCACGTGTTACTCACCCGTCCGCCGCTAACATCAGGGAGCAAGCTCCCATCTGTCCGCTCGACTGCA
