# Supplementary material for: Contribution of pectin-degrading bacteria to the quality of cigar fermentation: an analysis based on microbial communities and physicochemical components
Source: Front Microbiol. 2024 Nov 14;15:1481158. doi: 10.3389/fmicb.2024.1481158 (PMC11604125; doi:10.3389/fmicb.2024.1481158)
Supplement: Supplementary file 6 [file Table_2.DOCX]

**Response to Reviewers and Editors**

Dear Editors and Reviewers,

We are sincerely grateful for the valuable suggestions and comments provided by the reviewers and editorial board. We have carefully reviewed each suggestion and made detailed revisions to the manuscript accordingly. All revisions have been made in track changes mode for clarity. Below is a point-by-point response to each of the suggestions and the corresponding modifications.

**Response to Reviewer 1：**

1. Suggestion：What exactly is the NA medium?

response or modification：Nutrient Agar (NA) is a commonly used basic medium in microbiology, especially for the isolation and cultivation of bacteria. It is the solid version of Nutrient Broth (NB), solidified by adding agar, making it suitable for experiments such as colony morphology observation and quantitative analysis. The main components are beef extract: 3.0 g, peptone: 5.0 g, sodium chloride: 5.0 g, agar: 15.0 g, and distilled water: 1 L.

2. Suggestion：Could you elaborate a bit more on the screening methodology?

response or modification：In the first step, a large number of strains were screened from healthy tobacco leaves (including typical brands from Yunnan, Dominican Republic, and Cuba) using the dilution spread plate method. In the second step, pectinolytic strains were selected using a pectinase-producing identification medium, primarily based on the presence of halos. The hydrolysis capacity value (HC value), which is the ratio of halo diameter to colony diameter, was used to assess enzyme production capacity. A larger ratio indicates a stronger enzyme-producing ability, and the best candidate strains were determined based on this value.

3. Suggestion：Please abbreviate acronyms and abbreviations for the first time they appear in the full form within the manuscript.

response or modification：I appreciate the reviewer’s feedback in pointing out the errors, and I have made the necessary corrections.

4. Suggestion：Some figure captions could be more detailed. It is recommended to capitalize the first letter of "countdown" in Figure 1.

response or modification：I appreciate the reviewers for pointing out the errors, and I have made the necessary corrections. The modifications are as follows:

**Response to Reviewer 2：**

1. Suggestion：The tenses in the abstract are confusing.

response or modification：Thank you to the reviewer for pointing out the tense errors in the manuscript. I have revised the abstract accordingly. If there are still any issues, I kindly ask the reviewer to point them out, and I will make further revisions.

2. Suggestion：There is some confusion in the singular and plural usage of “tobacco leaf” and “microbial communities”.

response or modification：I appreciate the reviewers for pointing out the errors, and I have made consistent modifications throughout the manuscript regarding singular and plural forms.

3. Suggestion：The size of the hydrolysis zone is limited by the diffusion ability of substances in agar. The author should specifically measure the pectinase activity.

response or modification：Following the reviewer’s suggestion, the pectinase activity of the two bacterial strains was specifically measured. The results are as follows:

4. Suggestion：In Line 123, 148, and Figure 1, the pectin-degrading bacteria were screened at 28°C, why cigar leaves were fermented at 35°C?

response or modification：The temperature and humidity set for this fermentation experiment were designed to simulate the actual industrial fermentation conditions of the Modu Cigar Tobacco Drying and Fermentation Management Workshop in Ede, Mosha Town, Xinping County, Yuxi City, Yunnan Province, China. In the local fermentation facility, the temperature is consistently set to 35°C and the humidity to 75%. The incubation temperature for the bacterial strains was set at 28°C because experiments showed that the strains proliferated more effectively at this temperature, with higher OD600 values compared to other temperatures, indicating a larger bacterial population. Moreover, the strains exhibited higher activity at 28°C. Therefore, incubating the strains at 28°C allows for more stable and significant metabolic activity during the actual fermentation process, which in turn enhances the fermentation quality.

5. Suggestion：In Figure 1, it should be“YX-2” instead of “YX-1”. Besides, the part of “sensory evaluation” should not belong to the part of “Physicochemical composition”.

response or modification：Thank you very much to the reviewer for identifying this critical error. I have made the necessary corrections, and I have also accepted the reviewer’s suggestion that sensory evaluation does not fall under physicochemical properties, and this has been revised accordingly. The modifications are as follows:

6. Suggestion：For sensory evaluation, bitterness is insufficient to show that tobacco quality has improved. Aroma, irritant, and other index should also be included.

response or modification：Thank you to the reviewer for the insightful question! The Modu Cigar Tobacco Drying and Fermentation Management Workshop in Ede, Mosha Town, Xinping County, Yuxi City, Yunnan Province, is an important cigar tobacco fermentation facility in Yunnan Province. Long-term fermentation practices by technical personnel from the Yuxi Tobacco Company have revealed that cigar tobacco fermented in this region tends to have a higher pectin content, which contributes to a stronger green bitterness. Therefore, this experiment was conducted with the aim of reducing the pectin content in cigar tobacco. The focus of this study was to examine the pectin content and green bitterness of cigar tobacco, alongside key physicochemical properties such as total nitrogen, total sugar, and reducing sugar, all of which are crucial indicators of cigar quality. Additionally, we measured the total aroma components, which directly reflect the aroma quality of the cigar tobacco.

7. Suggestion：How were the fermentation conditions for cigar leaves (such as temperature and humidity) determined, and are they the optimal fermentation conditions? The fermentation time of cigar leaves in this study was 35 days, which is much longer than the fermentation time of most literature reports on cigar leaves. Is the fermentation time too long?

response or modification：The temperature and humidity settings for this fermentation experiment were designed to simulate the actual industrial fermentation conditions at the Modu Cigar Tobacco Drying and Fermentation Management Workshop in Ede, Mosha Town, Xinping County, Yuxi City, Yunnan Province, China. The local fermentation facility maintains a constant temperature of 35°C and a humidity level of 75%. These conditions were chosen to closely replicate the actual fermentation environment in order to evaluate the contribution of the bacterial strains to the quality of cigar tobacco during the fermentation process. The fermentation duration at this facility varies depending on the tobacco variety. For the "Yunxue No.1" cigar tobacco used in this experiment, the required fermentation period ranges from 32 to 40 days. To facilitate sample collection, turning, and record-keeping, we used a weekly cycle (7 days) as a unit of measurement. After discussions with the technical staff from Yuxi Tobacco Company, the total fermentation perio*Candida* autochthonous starter for cigar fermentation via dissecting the microbiome. *Frontiers in Microbiology*, 14, 1138877.

8. Suggestion：Why was the growth curve of YX-2 and DM-3 not measured? Most Bacillus has reached the decline stage after 48 h. Why choose microorganisms at this time point for fermentation? Is it possible that the 48-hour culture has caused YX-2 and DM-3 to enter the declining phase, leading to a lower number of live bacteria on the wrapper leaf ?

response or modification：In the preliminary stages of the experiment, we conducted growth curves for the two bacterial strains. However, as these were not key parameters for this experiment, they were not included in the final data set. During the experiment, we observed that a 48-hour incubation period was optimal for the activity of strains YX-2 and DM-3. Although most *Bacillus* strains may enter the decline phase after 48 hours, based on the reviewer’s suggestion, we added enzyme activity measurements for both strains at 24, 36, and 48 hours of incubation. The results showed that both strains exhibited higher enzyme activity and metabolic capacity at the 48-hour mark. Therefore, this time point was selected as the optimal incubation period for fermentation.

9. Suggestion：When microbiological data is uploaded to NCBI, a specific data link should be given.

response or modification：Thank you for the reviewer's suggestion. Since this data is part of a collaborative project with the Tobacco Company and is covered under a confidentiality agreement, I will consult with the company to determine whether the data can be uploaded and a link provided.

10. Suggestion：Specific inoculum information should be given in cigar fermentation experiments.

response or modification：Thank you for the reviewer's suggestion. The specific inoculant information has now been added accordingly.

specific inoculants as follows: pure water, 1×10^-8^ CFU/mL YX-2 fermentation broth, and 1×10^-8^ CFU/mL DM-3 fermentation broth.

11. Suggestion：In Figure 4, why are the units of flavor substances 4 mol/L and no error bars provided?

response or modification：The total aroma components in this study were determined using GC-MS, which identified thousands of compounds in the tobacco leaves. These compounds were categorized based on the type of product they belong to, including chlorophyll degradation products, carotenoid degradation products, Maillard reaction products, and cembranoid degradation products. The unit used is mol/L, as the concentration of each individual compound detected is expressed in mol/L, and therefore, when grouped, the same unit is used. The GC-MS analysis was conducted by a specialized company with three replicates, and the data presented are averages. Consequently, no standard deviations are provided, as each aroma component category is derived from the grouping of dozens of individual compounds.

12. Suggestion：In 3.3 and 3.4, with regard to the effect of pectin-degrading bacteria on fungal diversity, it seems that the conclusions drawn in Figure 6 and Figure 7 are contradictory.

response or modification：I appreciate the reviewers for raising the professional issues. In Figure 6, the Venn diagram and stacked bar chart mainly illustrate the species composition and shared species across different groups. It focuses on the differences in species diversity and types among the treatment groups. Specifically, the Venn diagram clearly shows the common and unique fungal genera between groups, as well as the specific impact of fermentation treatment on the microbial community composition. In contrast, the Alpha diversity graph in Figure 7 displays the species diversity and evenness within each treatment group, focusing on community structural characteristics, such as species richness (Sobs index) and community evenness (Simpson and Shannon indices). Therefore, the two figures emphasize different aspects: the Venn diagram highlights species presence, while the Alpha diversity analysis focuses on species richness and distribution evenness. Even if the Venn diagram shows minimal differences in species composition, the Alpha diversity indices may reveal significant variations due to differences in species abundance and distribution.

Furthermore, the Venn diagram reveals the number of species and their classification differences among the three treatment groups, but it does not directly show the distribution of species within each group. The Venn diagram merely presents the presence or absence of species without reflecting their relative abundance or community evenness. However, the Simpson and Shannon indices in the Alpha diversity analysis depict species evenness. Even if the number of species is similar, the presence of dominant species may cause the Simpson index to decrease, indicating uneven species distribution within the community. Although the TDM3 group shows higher diversity in both the Sobs index (species richness) and the Venn diagram, the Simpson index suggests that dominant species may have reduced evenness within the community. Thus, even though the Venn diagram indicates that the TDM3 group has the highest number of species, this does not necessarily imply high community evenness. On the contrary, according to the Simpson index, certain dominant species may lead to uneven distribution within the community. Both figures reflect different characteristics of the same community. Additionally, Figure 7 shows that the TDM3 group exhibits higher diversity in the Sobs index (species richness) and the Venn diagram, suggesting that it may contain more fungal genera. However, the Simpson index shows decreased community evenness in the TDM3 group, indicating that while there are more species, certain species dominate the community. This is consistent with the fact that the Venn diagram reflects species richness but does not directly show evenness. This demonstrates that although the TDM3 group has a higher number of species (as shown by the Venn diagram and Sobs index), its community may be dominated by specific species, resulting in reduced evenness (as indicated by the Simpson index). This phenomenon is common in diversity studies and does not indicate contradictory conclusions but rather reflects different aspects of diversity research. Therefore, the data itself is not contradictory; however, due to inappropriate descriptions by the author, I have made revisions accordingly.

13. Suggestion：In 3.5, the title is “Effect of adding pectin-degrading bacteria on species differences in cigar tobacco leaves”, however, only the differences on genus were shown in Figure 8 and Figure 9.

response or modification：Thank you to the reviewer for pointing out the error. This study focuses on describing community differences at the genus level because it provides a balance between information richness and interpretability. Compared to species-level analysis, genus-level descriptions can capture the major functional characteristics of the community, as microbes within the same genus often share similar metabolic or ecological functions. This level of description is sufficient for explaining the ecological roles of microorganisms. Moreover, genus-level analysis is technically more feasible. Common identification techniques, such as 16S rRNA sequencing, offer high resolution and accuracy at the genus level, whereas at the species level, precise identification often faces technical limitations due to high genetic sequence similarity. As a result, many researchers prefer genus-level descriptions, especially when dealing with large-scale microbial community studies. In addition, in the context of cigar tobacco fermentation or food fermentation, the focus is usually on the functional characteristics of specific genera rather than the details of species. Genus-level analysis provides sufficient information to help researchers identify community functions and environmental adaptability without the need to delve into the finer classification of species or strains.

14. Suggestion：Figure 6 and Figure 9, since YX-2 and DM-3 have been identified as Bacillus flexus and Bacillus siamensis, respectively, but the abundance of Bacillus is quite low, whether the two strains were still viable at 35 days? How can it be proved that these two strains degraded pectin?

response or modification：Thank you to the reviewer for raising this insightful question, which provides direction for our future exploration. As the reviewer pointed out, our team had similar doubts at the beginning. Therefore, we conducted an additional treatment group using Bacillus subtilis, a strain without pectin-degrading ability, for fermentation. We found that the pectin content in this group was comparable to the CK group, with no significant difference. In contrast, the pectin content in the groups fermented with the two pectin-degrading strains, as shown in the data, decreased significantly. This indicates that these two strains play a notable role. Regarding their survival over 35 days, this is indeed a critical point that requires further attention. As our study primarily focused on tobacco leaf quality and the extent of pectin degradation, this aspect was overlooked, which I will address in the discussion section to highlight the limitations of this study. The primary focus of this research was to measure the significant reduction in pectin content following fermentation with pectin-degrading bacteria. The degradation products, such as total sugars, also increased. Furthermore, the sensory evaluation conducted by cigar experts confirmed that the addition of these two strains reduced pectin content, improving the taste quality. In order to better understand the relationship between microbial community changes and tobacco leaf quality, we analyzed the key microbial differences after the addition of pectin-degrading bacteria. This analysis helps to reveal the advantages of these strains in cigar tobacco fermentation.

15. Suggestion：The author should specifically analyze the aroma substances with significant differences to better illustrate the role of microorganisms.

response or modification：Thank you for the reviewers' suggestions. I have made revisions accordingly.

16. Suggestion：Why is there no CK group in Figure 8B?

response or modification：Figure 8B does not include the CK group because, during the plotting analysis, no significant differences were observed between the groups, resulting in the control group (CK) not being displayed. In certain cases, if the LDA score or abundance differences between the control and treatment groups are not significant, displaying these differences lacks practical relevance. Therefore, the CK group was omitted in the figure based on the analysis results.

17. Suggestion：There is a problem with the legend in Figure 12, please check carefully.

response or modification：In the legend, red lines indicate negative correlations between taxa, while green lines represent positive correlations. The node colors correspond to different genera, such as p_Ascomycota, where "p" stands for the phylum and the name following it is the genus, which should be italicized, while "p" remains in regular font. I have made the necessary revisions accordingly.

18. Suggestion：In the discussion, the author only discussed the positive effect of Pseudomonas on pectin degradation. Compared with the effects of other microbial fermentation of cigar leaves reported in literatures, what are the advantages of fermenting cigar leaves with pectinolytic bacteria in this study? More discussion needed here. Discussions are also needed for other bacterial and fungal microorganisms with significant changes.

response or modification：Accepting the reviewer’s suggestion, we further explore the advantages of pectin-degrading bacteria in the fermentation of cigar tobacco leaves and conduct further discussion on other bacterial and fungal microorganisms that showed significant changes.

We sincerely thank the editors and reviewers for their thorough review and valuable suggestions on our manuscript. Following your recommendations and requirements, we have made comprehensive revisions and also corrected some inaccuracies identified during the process. We deeply appreciate the errors you pointed out and the constructive feedback you provided, which have greatly enhanced the integrity and quality of our paper. We warmly welcome any further suggestions for improvement and eagerly look forward to the opportunity of having our article published in your esteemed journal!

Best regards,

Youbo Su, Xueru Song

October 22, 2024
